# A pilot study to evaluate the effect of CT1812 treatment on synaptic density and other biomarkers in Alzheimer’s disease

**DOI:** 10.1186/s13195-024-01382-2

**Published:** 2024-01-25

**Authors:** Christopher H. van Dyck, Adam P. Mecca, Ryan S. O’Dell, Hugh H. Bartlett, Nina G. Diepenbrock, Yiyun Huang, Mary E. Hamby, Michael Grundman, Susan M. Catalano, Anthony O. Caggiano, Richard E. Carson

**Affiliations:** 1 Alzheimer’s Disease Research Unit, Department of Psychiatry, Yale University School of Medicine, New Haven, CT USA; 2 Department of Radiology and Biomedical Imaging, Yale University, New Haven, CT USA; 3 Cognition Therapeutics Inc., Pittsburgh, PA USA; 4 Global R&D Partners, LLC, San Diego, CA USA; 5 Department of Neurosciences, University of California, San Diego, USA; 6 Circle Biopharma, Pittsburgh, PA USA

**Keywords:** Alzheimer’s disease, Sigma-2 receptor, Amyloid beta oligomers, SV2A PET, FDG PET, Biomarkers

## Abstract

**Background:**

Effective, disease-modifying therapeutics for the treatment of Alzheimer’s disease (AD) remain a large unmet need. Extensive evidence suggests that amyloid beta (Aβ) is central to AD pathophysiology, and Aβ oligomers are among the most toxic forms of Aβ. CT1812 is a novel brain penetrant sigma-2 receptor ligand that interferes with the binding of Aβ oligomers to neurons. Preclinical studies of CT1812 have demonstrated its ability to displace Aβ oligomers from neurons, restore synapses in cell cultures, and improve cognitive measures in mouse models of AD. CT1812 was found to be generally safe and well tolerated in a placebo-controlled phase 1 clinical trial in healthy volunteers and phase 1a/2 clinical trials in patients with mild to moderate dementia due to AD. The unique objective of this study was to incorporate synaptic positron emission tomography (PET) imaging as an outcome measure for CT1812 in AD patients.

**Methods:**

The present phase 1/2 study was a randomized, double-blind, placebo-controlled, parallel-group trial conducted in 23 participants with mild to moderate dementia due to AD to primarily evaluate the safety of CT1812 and secondarily its pharmacodynamic effects. Participants received either placebo or 100 mg or 300 mg per day of oral CT1812 for 24 weeks. Pharmacodynamic effects were assessed using the exploratory efficacy endpoints synaptic vesicle glycoprotein 2A (SV2A) PET, fluorodeoxyglucose (FDG) PET, volumetric MRI, cognitive clinical measures, as well as cerebrospinal fluid (CSF) biomarkers of AD pathology and synaptic degeneration.

**Results:**

No treatment differences relative to placebo were observed in the change from baseline at 24 weeks in either SV2A or FDG PET signal, the cognitive clinical rating scales, or in CSF biomarkers. Composite region volumetric MRI revealed a trend towards tissue preservation in participants treated with either dose of CT1812, and nominally significant differences with both doses of CT1812 compared to placebo were found in the pericentral, prefrontal, and hippocampal cortices. CT1812 was safe and well tolerated.

**Conclusions:**

The safety findings of this 24-week study and the observed changes on volumetric MRI with CT1812 support its further clinical development.

**Trial registration:**

The clinical trial described in this manuscript is registered at clinicaltrials.gov (NCT03493282).

**Supplementary Information:**

The online version contains supplementary material available at 10.1186/s13195-024-01382-2.

## Introduction

Although approximately 6 million people in the US suffer from Alzheimer’s disease (AD), the precise mechanism of disease is poorly understood, and development of disease-modifying therapeutics (DMTs) remains a significant challenge [1]. Amyloid beta (Aβ) is a key target for drug development based on extensive evidence of its involvement in the synaptic dysfunction and loss central to AD pathophysiology [2]. Several convergent lines of evidence have suggested that Aβ oligomers are the most toxic form of the protein [3]. The binding of Aβ oligomers to their receptors on neurons is followed by reductions in synaptic protein expression, spine and synapse number, as well as by the loss of synaptic plasticity and failure of new memory formation [4–6]. Anti-Aβ monoclonal antibodies in development as DMTs have limited selectivity for Aβ oligomers, and their use is associated with adverse events (AEs) including amyloid-related imaging abnormalities (ARIA) [7].

CT1812 is a novel brain penetrant molecule that interferes with the binding of Aβ oligomers to neurons, preventing synaptotoxicity [8, 9]. More specifically, CT1812 binds to the sigma-2 receptor and allosterically displaces Aβ oligomers that are bound to neurons. CT1812 dose-dependently displaces Aβ oligomers into the cerebrospinal fluid (CSF) and improves cognitive performance in mouse models of AD [8]. Early interventional clinical trials studying CT1812 in patients with AD have shown evidence of changes in disease-related biomarkers including increased levels of CSF Aβ oligomers [10], reduced levels of CSF synaptic proteins and phosphorylated Tau (pTau), and normalized abundance of other AD-related proteins [8].

CT1812 was generally safe and well tolerated in more than 80 healthy volunteers in a phase 1 placebo-controlled safety study (COG0101; NCT02570997) [11]. In a subsequent phase 1a/2 placebo-controlled trial (COG0102; NCT02907567), 19 subjects with mild to moderate AD were safely administered CT1812 for 28 days; all three dose levels (90, 280 and 560 mg/day) were well tolerated and were not associated with any serious AEs (SAEs). All AEs were mild or moderate in nature. Given the short duration of the study, there was no expectation to see improvements in the exploratory cognitive outcomes, however, it is notable there were no differences or acute worsening in any of the treatment groups.

With the recent advent of synaptic positron emission tomography (PET) imaging, we have begun to evaluate synaptic alterations in vivo. Synaptic vesicle glycoprotein 2A (SV2A) is expressed in virtually all synapses and is located in synaptic vesicles at presynaptic terminals [12]. [^11^C]UCB-J was recently developed as a PET tracer for SV2A and advanced for human studies [13]. In our recent cross-sectional studies of [^11^C]UCB-J PET in AD, we observed widespread reductions of SV2A binding in medial temporal and neocortical brain regions in early AD compared to cognitively normal participants [14, 15]. Moreover, [^11^C]UCB-J PET was highly correlated with cognitive performance, suggesting that synaptic imaging might be a potential surrogate biomarker outcome for therapeutic trials that is well-correlated with clinical measures [16].

The present phase 1/2 study (COG0105; NCT03493282) was a randomized, double-blind, placebo-controlled, parallel-group trial conducted in participants with mild to moderate dementia due to AD to evaluate the safety, efficacy, pharmacokinetics (PK), and pharmacodynamics (PD) of CT1812 over 6 months with a 6-month optional extension. The primary objective was to determine safety and tolerability in patients with AD. Secondary objectives evaluated the effect of CT1812 on canonical AD and synaptic biomarkers, along with synaptic density, and cognitive and clinical outcomes in the six-month primary study. Here, we report the SV2A and fluorodeoxyglucose (FDG) PET, volumetric MRI, CSF biomarker, and clinical findings from this pilot study.

## Methods

### Study protocol

This study protocol was reviewed and approved by the Yale Human Investigation Committee and Yale Radiation Safety Committee. All study participants and their study partners provided written informed consent to participate in this study. Because, to this date, there has not been evidence of clinical benefit resulting from short treatment durations such as the one in this phase 1/2 study, participants were required to provide informed consent for themselves (that is, surrogate consent was not permitted).

### Objectives and outcome measures

The primary objective of this study was to evaluate the safety and tolerability of CT1812 in patients with AD. Secondary objectives were to evaluate the effect of CT1812 on brain synaptic density using the SV2A PET ligand [^11^C]UCB-J and on brain metabolic activity using FDG PET; on cognitive and clinical outcomes using the Alzheimer’s Disease Assessment Scale – cognition subscale 11 (ADAS-Cog11) and ADAS-Cog13 (derived from ADAS-Cog14), the Alzheimer’s Disease Cooperative Study—Activities of Daily Living (ADCS-ADL), the mini Mental State Exam (MMSE), and the Clinical Dementia Rating Scale Sum of Boxes (CDR-SB); on brain volume using volumetric MRI; and on CSF biomarkers associated with AD including Aβ40, Aβ42, total Tau, pTau, neurogranin, synaptosomal-associated protein-25 (SNAP-25), synaptotagmin, and neurofilament light (NfL). Additional secondary and exploratory objectives and outcome measures are not reported in this manuscript.

### Participants and inclusion criteria

Participants included men and women of non-childbearing potential (50–85 years of age) with a diagnosis of mild to moderate dementia due to AD and at least 6 months of cognitive decline, an MRI consistent with AD diagnosis, a MMSE score of 18–26, and either a positive amyloid PET scan or a positive CSF biomarker test consistent with AD. Participants were excluded based on hospitalization or change of chronic concomitant medication within one month prior to screening, a significant abnormality detected on screening MRI (e.g., prior hemorrhage or infarct > 1 cm^3^, > 3 lacunar infarcts, cerebral contusion, encephalomalacia, aneurysm, vascular malformation, subdural hematoma, hydrocephalus, or space-occupying lesion), another primary cause of dementia (such as dementia with Lewy bodies, frontotemporal dementia, Huntington’s disease, Creutzfeldt-Jakob disease, or Down’s syndrome), any significant neurological disease (such as Parkinson’s disease, multiple sclerosis, stroke, seizure disorders, or other infectious, metabolic, or systemic diseases that impact the central nervous system), diagnosed depression (unless successfully managed by antidepressants), schizophrenia or bipolar disorder, and other unstable medical conditions. Also excluded were participants who had received an anti-Aβ monoclonal antibody or BACE inhibitor within 180 days or who had previously received an Aβ-targeting vaccine.

### Study drug administration

Following completion of the baseline procedures, the first dose of study medication or placebo was administered in the clinic and participants were observed for two hours post-dose. Study medication included CT1812 at 300 or 100 mg or placebo, provided as two hydroxypropyl methylcellulose capsules containing CT1812 fumarate salt or lactose monohydrate (placebo). The participants were instructed to take study drug at home each morning with or without food, except on clinic days, when the study drug was taken during the visit. Study treatment was administered orally as a single daily dose (two total capsules per day) for 24 weeks. Participants returned to the clinic for repeat psychometric/neurologic testing, safety studies, and PK and PD sample collection approximately every 2 weeks for the first 6 weeks and then every 3 weeks thereafter for the duration of the study.

### Safety and tolerability endpoints

The safety and tolerability of CT1812 were assessed by collecting vital signs, 12-lead ECGs, physical and neurological examination data, clinical laboratory test results (including hematology, serum chemistry, coagulation, and urinalysis-related tests), Columbia-Suicide Severity Rating Scale (C-SSRS), and adverse event (AE) monitoring. Blood samples were collected for analysis of CT1812 concentrations in plasma prior to administration of study drug on the day of collection.

### Clinical efficacy measures

The ADAS-Cog14 is a widely used general cognitive measure in clinical trials of AD that assesses multiple cognitive domains including memory, language, praxis, and orientation [17]. A higher score indicates more impairment, a positive change indicates cognitive worsening. The ADAS-Cog11 subscale was the primary efficacy endpoint of this clinical trial.

The ADCS-ADL is a 23-item questionnaire developed by the Alzheimer’s Disease Cooperative Study (ADCS) to assess the ability to perform activities of daily living (ADLs) by participants with AD [18]. The ADCS-ADL scale discriminates well between cognitively normal participants and those with AD and it has good test–retest reliability. The scale ranges from 0–78 and a lower score indicates more impairment.

The MMSE is a brief screening instrument, often used in clinical trials to assess dementia severity, and measures several aspects of memory and cognitive functioning including orientation, attention, concentration, comprehension, recall, and praxis [19]. The scoring range is 0–30, and a lower score indicates more cognitive impairment. The MMSE was administered at screening to determine eligibility for the trial as well as post-dose.

The CDR-SB Scale is a clinician-rated dementia staging system that tracks the progression of cognitive and functional deterioration [20]. It includes semi-structured interviews with both participants and their caregivers and assesses cognition and function across six domains (memory, orientation, judgement and problem-solving, community affairs, home and hobbies, and personal care). Scores for each domain range from 0 to 3 while total scores range from 0 to 18, with higher scores indicative of greater impairment.

All assessments were performed at baseline and 6, 12, 18 and 24 weeks.

### Brain imaging

T1-weighted magnetic resonance imaging (MRI) was performed at baseline, 12 weeks, and 24 weeks to define regions of interest (ROI) and to assess brain volumetric changes. T1 MRI acquisition parameters included a Sag 3D magnetization-prepared rapid gradient-echo (MPRAGE) sequence with 2.95-ms echo time, 2300-ms repetition time, 900-ms inversion time, 9° flip angle, and 240 Hz/pixel bandwidth. Images are 256 × 256 × 176 with a pixel size of 1.1 × 1.1 × 1.2 mm. Additional clinical sequences were performed to ensure that participants did not meet any exclusion criteria at screening and did not develop any treatment emergent adverse events, including amyloid related imaging abnormalities (ARIA). These sequences included axial T2-star gradient echo (GRE), axial T2 fluid attenuated inversion recovery (FLAIR), axial diffusion weighted imaging (DWI), and axial T2 fast/turbo spin echo (FSE/TSE). MRI was collected on a 3 T Trio (Siemens Medical Systems, Erlangen, Germany) with a circularly polarized head coil.

PET scans were performed on the high-resolution research tomograph (HRRT) (207 slices, resolution ~ 3 mm full width half maximum) [21], with list-mode data reconstructed using the MOLAR algorithm [22] and event-by-event motion correction based on an optical detector (Vicra, NDI Systems, Waterloo, Canada, [23]).

All participants received a Pittsburgh Compound B ([^11^C]PiB) PET scan during screening to determine the presence or absence of brain Aβ accumulation [15, 24–26] and were required to be Aβ positive by both visual and quantitative criteria. Visual criteria were evaluated by an experienced reader, and quantitative criteria required a [^11^C]PiB cerebral-to-cerebellar distribution volume ratio (*DVR*) of 1.40 or more in at least 1 AD-affected ROI [15, 24]. Dynamic [^11^C]PiB scans were acquired for 90 min following administration of a bolus of up to 555 MBq (15 mCi) of tracer.

All participants also received three [^11^C]UCB-J PET scans (baseline, 12 weeks, and 24 weeks) and two [^18^F]FDG PET scans (baseline and 24 weeks) throughout the course of this study. Dynamic [^11^C]UCB-J scans were acquired for up to 90 min after administration of a bolus of up to 740 MBq (20 mCi) [27], and dynamic [^18^F]FDG scans were acquired for up to 90 min after administration of a bolus of up to 185 MBq (5 mCi) [28]. Participants were required to fast for at least 4 h prior to the [^18^F]FDG PET scan. Dynamic [^11^C]UCB-J and [^18^F]FDG images were motion corrected using a mutual-information algorithm (FSL-FLIRT; frame-by-frame registration to a summed 0–10 min image) and then registered to each participant’s MRI.

### Tracer kinetic modeling

For [^11^C]UCB-J image analysis, parametric images of *BP*_ND_ were generated using a simplified reference tissue model (SRTM2) from 0 to 60 min [29] and a small ROI (2 mL) in the core of the centrum semiovale as the reference region [30, 31]. As previously described, values of *DVR* using a whole cerebellum reference region were then computed for each voxel as (*BP*_ND_ + 1)/(*BP*_ND_[cerebellum] + 1) [15, 32]. For [^18^F]FDG image analysis, parametric images of standardized uptake value (*SUV*) were generated by summing radioactivity concentration from 60 to 90 min. Values of *SUVR* using a whole cerebellum reference region were then computed for each voxel as *SUV*/*SUV* [cerebellum]. Partial volume correction was not applied to these images.

### Regional analyses

Longitudinal analysis of change in brain gray matter volume was conducted by first performing cortical reconstruction and volumetric segmentation at individual timepoints for each participant using FreeSurfer (version 6.0) [33]. ROIs from the FreeSurfer cortical parcellation and subcortical segmentation were used for both PET and MRI analyses performed in native subject space. Cortical regions were defined by the Desikan-Killiany atlas [33, 34]. To elucidate the effects of CT1812 on imaging outcomes, a composite ROI of AD-affected brain regions was defined (Supplemental Table 1). Individual exploratory brain regions included hippocampus, entorhinal cortex, parahippocampal cortex, amygdala, fusiform gyrus, lingual gyrus, inferior/middle temporal cortex, anterior cingulum, posterior cingulum, precuneus, prefrontal cortex, superior temporal cortex, lateral parietal cortex, lateral occipital cortex, pericentral cortex, and medial occipital cortex (Supplemental Table 2). For each time point, MRI determined gray matter volume was normalized using estimated intracranial volume [35] and changes from baseline were assessed using mixed models for repeated measures as described in the Statistical Methods.

**Table 1:** Baseline participant characteristics

	Placebo (*N* = 7)	CT1812 100 mg (*N* = 8)	CT1812 300 mg (*N* = 8)	Total (*N* = 23)
Age (years)	72.6 ± 5.8	68.0 ± 9.3	69.6 ± 10.9	70.0 ± 8.8
Sex	4 M, 3 F	4 M, 4 F	4 M, 4 F	12 M, 11 F
Race				
Black or African American	1 (14%)	-	-	1 (4%)
White	6 (86%)	8 (100%)	8 (100%)	22 (96%)
Ethnicity				
Hispanic or Latino	-	-	-	-
Not Hispanic or Latino	7 (100%)	8 (100%)	8 (100%)	23 (100%)
Weight (kg)	74.8 ± 9.2	85.6 ± 5.0	76.2 ± 16.9	79.0 ± 12.1
Height (cm)	172.1 ± 12.1	171.4 ± 12.5	170.3 ± 9.9	171.2 ± 11.0
BMI (kg/m^2^)	25.2 ± 1.8	29.5 ± 4.1	26.3 ± 5.7	27.1 ± 4.5
Education level achieved				
High school or GED	2 (29%)	-	1 (13%)	3 (13%)
College or some college	2 (29%)	5 (63%)	7 (88%)	14 (61%)
Postgraduate	3 (43%)	3 (38%)	-	6 (26%)
ApoE status				
APOE4 carrier	6 (86%)	7 (88%)	5 (63%)	18 (78%)
APOE4 non-carrier	1 (14%)	1 (13%)	3 (38%)	5 (22%)
MMSE (Total score)	22.3 ± 1.98	22.6 ± 1.51	22.9 ± 2.42	22.6 ± 1.92
Use of AD drugs				
ACHEI	6 (86%)	7 (88%)	7 (88%)	20 (87%)
Memantine	3 (43%)	1 (13%)	3 (38%)	7 (30%)

*M* Male, *F* Female, *neg* Negative, *pos* Positive, *MMSE* Mini mental state exam, *ACHEI* acetylcholinesterase inhibitor, ± standard deviation (SD)

**Table 2:** Safety results from primary 24 week study

	Placebo (*N* = 7)	CT1812 100 mg (*N* = 8)	CT1812 300 mg (*N* = 8)	Total (*N* = 23)
Number (%) of Subjects with TEAE [Number of TEAEs]				
TEAEs	5 (71%) [8]	8 (100%) [24]	5 (63%) [20]	18 (78%) [52]
Mild	3 (43%) [6]	6 (75%) [21]	4 (50%) [16]	13 (57%) [43]
Moderate	1 (14%) [1]	1 (12.5%) [1]	1 (13%) [4]	2 (9%) [6]
Severe	1 (14%) [1]	2 (25%) [2]	0 (0%)	3 (13%) [3]
Related TEAEs	3 (43%) [3]	3 (38%) [3]	4 (50%) [7]	10 (43%) [13]
TEAEs leading to treatment discontinuation	1 (14%) [1]	2 (25%) [2]	2 (25%) [2]	5 (22%) [5]
SAEs	1 (14%) [1]	2 (25%) [3]	0 (0%)	3 (13%) [4]
Related SAEs	0 (0%)	0 (0%)	0 (0%)	0 (0%) [0]
Number (%) of Subjects with Treatment-Related^a^ TEAE [Number of TEAEs]				
Headache	1 (14%) [1]	1 (13%) [1]	3 (38%) [3]	5 (22%) [5]
Dizziness	0 (0%)	1 (13%) [1]	1 (13%) [2]	2 (9%) [3]
Liver function test increase	0 (0%)	0 (0%)	2 (25%) [2]	2 (9%) [2]
Vomiting	1 (14%) [1]	1 (13%) [1]	0 (0%)	2 (9%) [2]
Diarrhea	1 (14%) [1]	0 (0%)	0 (0%)	1 (4%) [1]
Total related TEAEs	3 (43%) [3]	3 (38%) [3]	4 (50%) [7]	10 (43%) [13]

Top: Number and percentage of subjects experiencing an event and the total number of events in brackets for the six-month primary study period are summarized. The denominator for percentage corresponds to the N in each column. N is the number of subjects in the Safety Analysis Set. Bottom: AEs were coded using MedDRA version 21.0. TEAEs are events that occurred or worsened on or after the first application of study drug. Subjects are only counted once for each system organ class (SOC) and once for each preferred term (PT). The severity shown is the greatest severity reported for a particular subject. *TEAE* Treatment-emergent adverse event, *SAE* Severe adverse event; ^a^Related = possibly, probably, or definitely related

### Cerebrospinal fluid AD biomarkers

Each participant underwent a lumbar puncture (LP) as part of the screening process and prior to study drug administration at the Day 169 visit. Prior to the LP, a coagulation panel was obtained to rule out a clotting disorder. Participants were excluded from having an LP if they had an allergy to all local anesthetics (such as lidocaine) and/or had any medical condition requiring treatment with warfarin or heparin. CSF cell counts (white blood cells and red blood cells, with differential if either of the counts was abnormal), protein, and glucose were measured. CSF biomarkers of AD pathology and synaptic integrity were analyzed using validated methods for Aβ40, Aβ42, tau, and pTau with Lumipulse G1200 and G600 chemiluminescence enzyme immunoassay; neurogranin and NfL by ELISA; and SNAP-25 and synaptotagmin by IP-LC-PRM-MS.

### Statistical methods

Statistical analyses are described in detail in the Statistical Analysis Plan (Additional file 1). Briefly, characteristics of the participant dose groups were compared using χ2 test for categorical variables and unpaired t-tests for continuous variables. For all efficacy data (neuroimaging, clinical, and CSF), the change from baseline at 24 weeks was analyzed using a mixed model for repeated measures (MMRM), including fixed effects for treatment group, visit, and treatment*visit interaction. Baseline scores of each measure were included as covariates. In all primary analyses, treatment differences between CT1812 (pooling 300 mg and 100 mg doses) and placebo were estimated from the model for each visit. In addition, treatment differences between each of the dose groups and placebo at each visit were estimated. *P*-values and corresponding two-sided confidence intervals were provided.

Sample sizes of 7 per treatment group were chosen based on synapse loss estimations [36, 37] and were expected to yield a ~ 68% power for comparison of the pooled CT1812 treatment groups (*n* = 14) vs. 7 placebo group (*n* = 7, α = 0.05, 1-tailed test).

As per the statistical analysis plan, the full analysis set for efficacy includes all subjects who receive investigational product and who have at least one post-dose assessment of any of the cognitive and clinical endpoints.

For exploratory imaging analyses, no correction for multiple comparisons was made. All *p* values were nominal.

Safety data were summarized using descriptive statistics.

## Results

**Fig. 1:**
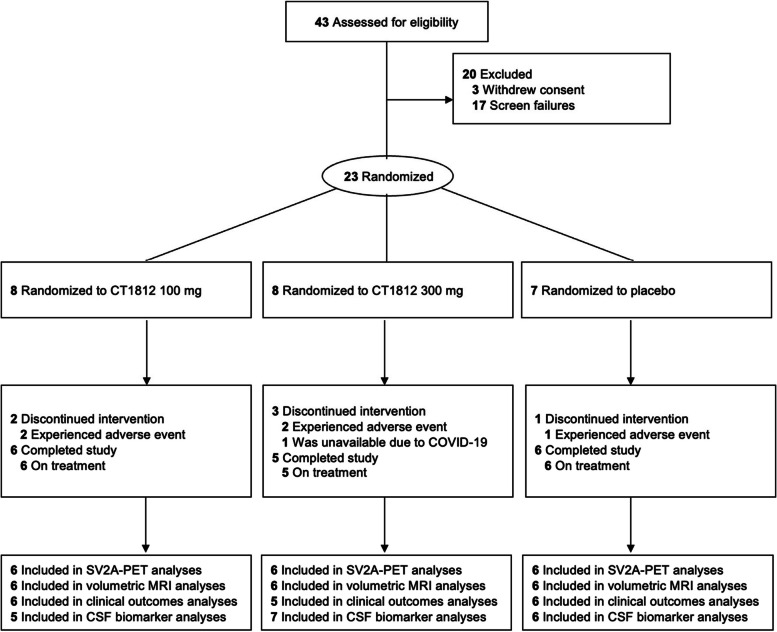
Study design CONSORT diagram. Forty-three participants were assessed for eligibility and 23 participants were randomized to receive placebo or 100 or 300 mg CT1812 once daily for 24 weeks. Two participants in the 100 mg and two in the 300 mg CT1812 group discontinued intervention due to AEs, and one in the 300 mg group became unavailable when pandemic-related travel restrictions precluded their return to the USA. One participant in the placebo group discontinued after experiencing an AE. PET was performed at baseline, 12 and 24 weeks to detect SV2A and at baseline and 24 weeks to detect FDG. MRI was performed and clinical outcomes were assessed at baseline, 12 and 24 weeks. CSF was sampled for biomarker assessments at baseline and 24 weeks. The participant numbers indicated for each assessment category are for the 24-week analysis

As shown in Fig. 1, a total of 43 participants were screened for this trial, and 23 were randomized: 7 were randomized to the placebo group, 8 to the CT1812 100 mg group, and 8 to the CT1812 300 mg group. All 23 participants were included in the safety analyses, and 17 (74%) completed the primary six-month study. Both the safety and the efficacy datasets included all participants that had been dosed with study drug. Early treatment discontinuations, primarily owing to adverse events, included 1 in the placebo group, 2 in the 100 mg group, and 3 in the 300 mg group. Compliance as assessed by remaining pill counts at each visit (mean percent, SD, min, max) was 95.5% (3.0, 91%, 100%), 88.7% (9.3, 74%, 100%) and 92.2% (7.7, 76%, 98%) for the 100 mg CT1812, 300 mg CT1812, and placebo groups, respectively.

### Participant characteristics

Baseline characteristics of the participant population are summarized in Table 1. These characteristics were generally well-balanced across the three treatment groups. The mean (SD) age was 70.0 (8.8) years; 52% of participants were male; 96% were White, and 4% were Black or African American. The mean (SD) body mass index (BMI) was 27.1 (4.5) kg/m^2^. The mean (SD) total MMSE score was 22.6 (1.92); 18 participants (78%) were *APOE* ε4 carriers and five (22%) were non-carriers.

### [^11^C]UCB-J and [^18^F]FDG PET

**Fig. 2:**
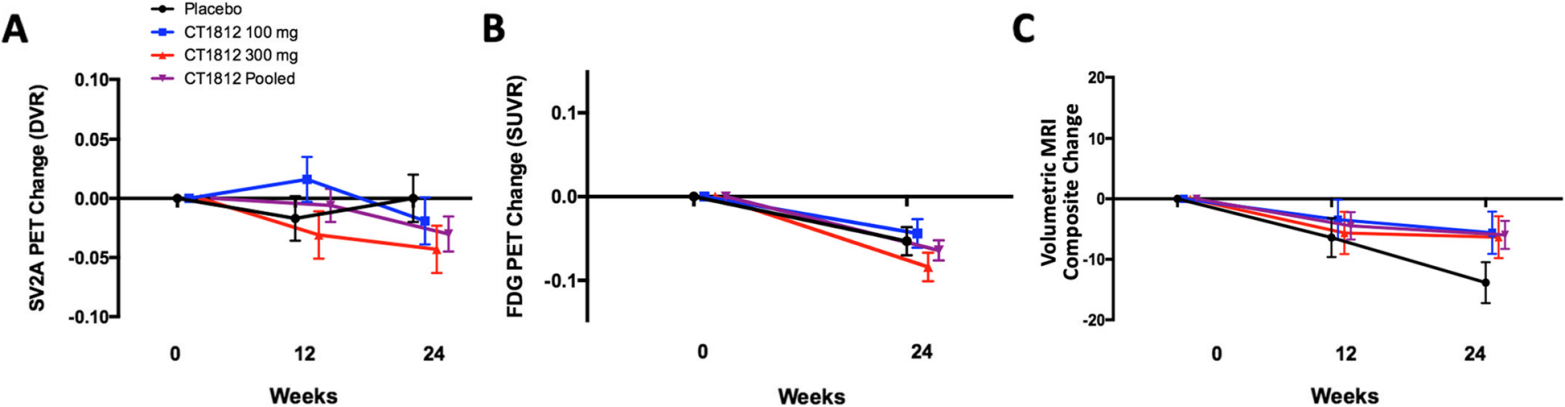
Neuroimaging measure changes from baseline through 24 weeks in composite region of interest. **A-B** PET was performed to detect SV2A at baseline, 12 and 24 weeks (**A**) and to detect FDG at baseline and 24 weeks (**B**). No statistical differences were observed between treatment groups in change from baseline for either SV2A or FDG. Actual change relative to the cerebellum reference region is shown; error bars represent SE values. **C** Subjects were assessed using MRI at baseline, 12, and 24 weeks. The mean change from baseline in volumetric MRI (mL) (error bars: SE) for the composite of brain regions showed a trend towards less volume loss for the combined dose groups vs. placebo

PET imaging results for [^11^C]UCB-J and [^18^F]FDG are displayed in Fig. 2. The primary imaging endpoint was the change from baseline at 24 weeks in synaptic density as measured by [^11^C]UCB-J *DVR* in the composite region in participants receiving CT1812 (with 300 mg and 100 mg doses pooled) versus placebo. The LS mean (SE) change from baseline, compared to change in the reference region (whole cerebellum), in [^11^C]UCB-J *DVR* composite region at 24 weeks was -0.030 (0.015) for the pooled CT1812 treatment groups, compared to 0.00 (0.020) for the placebo group. The LS mean (SE) difference from placebo for the pooled CT1812 treatment groups is -0.031 (0.025), with a 95% CI of -0.083 to 0.021 (*p* = 0.23). When dose groups were analyzed separately, no significant treatment differences were observed relative to placebo for the 100 mg group [-0.019 (0.028); 95% CI for difference from placebo: -0.078, 0.040; *p* = 0.51] or the 300 mg group [-0.043 (0.028); 95% CI for difference from placebo: -0.102, 0.015; *p* = 0.14] (Fig. 2A).

The effect of CT1812 on cerebral glucose metabolism, as a surrogate measure of neuronal and synaptic activity, was also evaluated using [^18^F]FDG PET. The LS mean (SE) change from baseline at 24 weeks in [^18^F]FDG PET SUVR in the composite region, compared to change in the reference region (whole cerebellum) was -0.064 (0.012) for the pooled CT1812 groups compared to -0.053 (0.017) for the placebo group. The LS mean (SE) difference from placebo for the pooled CT1812 treatment groups is -0.012 (0.022), with a 95% CI of -0.058 to 0.034 (*p* = 0.58). When dose groups were analyzed separately, no significant treatment differences were observed relative to placebo for the 100 mg group [-0.009 (0.24); 95% CI for difference from placebo: -0.043, 0.060; *p* = 0.72] or the 300 mg group [-0.032 (0.023); 95% CI for difference from placebo: -0.082, 0.019; *p* = 0.20] (Fig. 2B).

### Volumetric MRI

The effect of CT1812 on brain volume in AD participants was evaluated using volumetric MRI (Fig. 2C, Supplemental Table 3). A nonsignificant trend towards a treatment difference was observed for CT1812 relative to placebo in brain volume for the composite of brain regions. The LS mean (SE) change from baseline at 24 weeks in volumetric MRI in the composite region was -5.94 (2.34) for the pooled CT1812 groups compared to -13.85 (3.39) for the placebo group. The LS mean (SE) difference from placebo for the pooled CT1812 treatment groups was 7.86 (4.07), with a 95% CI of -0.50 to 16.21 (*p* = 0.06). When dose groups were analyzed separately, no significant treatment differences were observed relative to placebo for the 100 mg group [8.26 (4.98); 95% CI for difference from placebo: -2.01, 18.53; *p* = 0.11] or the 300 mg group [7.51 (4.81); 95% CI for difference from placebo: -2.40, 17.43; *p* = 0.13] (Fig. 2C). Exploratory region-specific analyses suggested a significant difference (more volume preservation) in the mean change from baseline at 24 weeks in the CT1812 treatment groups compared to placebo in the pericentral cortex, prefrontal cortex, and hippocampus (Supplemental Table 3). Other regions analyzed showed either a trend towards volume preservation or no change between CT1812 and placebo (Supplemental Table 3).

### Clinical outcomes

**Fig. 3:**
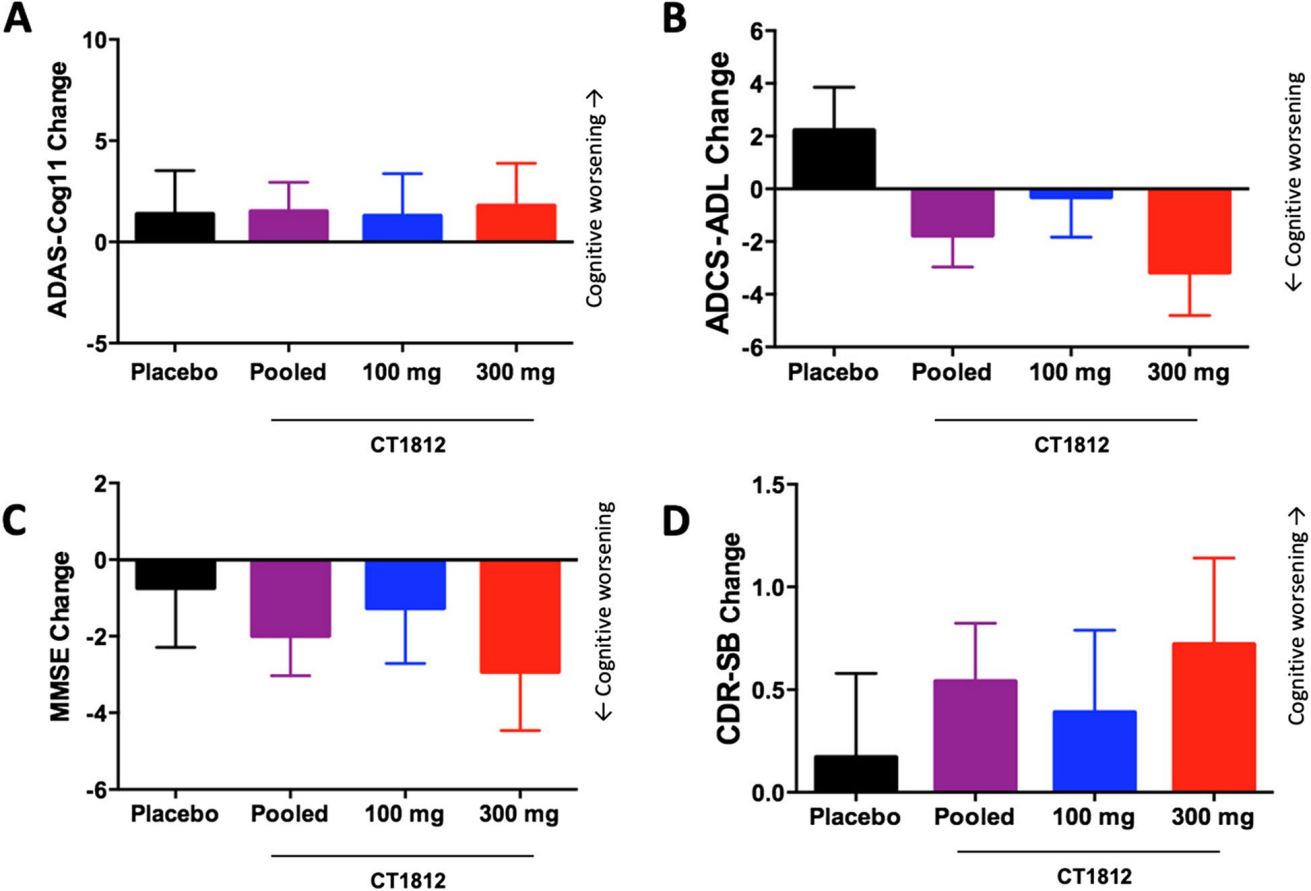
Change in clinical outcome measures through 24 weeks. Subjects were assessed using the ADAS-Cog11 (**A**), ADCS-ADL (**B**), MMSE (**C**), and CDR-SB (**D**) at baseline, 6, 12, 18 and 24 weeks. Data shown are LS Mean difference change (at 24 weeks) from baseline and SE from the linear mixed-effects model for repeated measures (MMRM). There were no differences throughout the 5 study visits. No significant differences were seen between any of the treatment groups in change from baseline through 24 weeks

Changes in cognitive function and clinical outcomes following CT1812 treatment were measured using the ADAS-Cog11, ADCS-ADL, MMSE, and CDR-SB clinical rating scales (Fig. 3). The LS mean (SE) change from baseline at 24 weeks on the ADAS-Cog11 rating scale was 1.50 (1.44) for the pooled CT1812 groups compared to 1.37 (2.14) for the placebo treatment group.

The LS mean (SE) difference from placebo for the pooled CT1812 treatment groups was 0.14 (2.54), with a 95% CI of -5.02 to 5.29 (*p* = 0.96). When dose groups were analyzed separately, no significant treatment differences were observed relative to placebo for the 100 mg group [-0.09 (2.99); 95% CI for difference from placebo: -6.18, 5.99; *p* = 0.98] or the 300 mg group [0.41 (3.01); 95% CI for difference from placebo: -5.69, 6.52; *p* = 0.89] (Fig. 3A).

The LS mean (SE) change from baseline at 24 weeks on the ADCS-ADL rating scale was -1.77 (1.19) for the pooled CT1812 groups compared to 2.21 (1.64) for the placebo treatment group. The LS mean difference from placebo for the pooled CT1812 treatment group was -3.99 (2.12) with a 95% CI of -8.25 to 0.26 (*p* = 0.07). When dose groups were analyzed separately, treatment differences relative to placebo were -2.52 (2.24) [95% CI -7.00, 1.97; *p* = 0.27] for 100 mg and -5.37 (2.33) [95% CI -10.03, -0.71; *p* = 0.02] for 300 mg (Fig. 3B).

The LS mean (SE) change from baseline at 24 weeks on the MMSE rating scale was -1.99 (1.04) for the pooled CT1812 groups compared to -0.74 (1.55) for the placebo treatment group. The LS mean difference from placebo for the pooled CT1812 treatment groups was -1.26 (1.85) with a 95% CI of -5.02 to 2.50; *p* = 0.50). When dose groups were analyzed separately, treatment differences relative to placebo were not significant for 100 mg [-0.52 (2.12); 95% CI for difference from placebo: -4.84, 3.80; *p* = 0.81] or for 300 mg [-2.18 (2.19); 95% CI for difference from placebo: -6.61, 2.26; *p* = 0.33] (Fig. 3C).

Finally, the LS mean (SE) change from baseline at 24 weeks on the CDR-SB rating scale was 0.54 (0.28) for the pooled CT1812 groups compared to 0.17 (0.41) for the placebo treatment group. The LS mean difference from placebo for the pooled CT1812 treatment groups was 0.37 (0.49) with a 95% CI for difference from placebo of -0.62, 1.37; *p* = 0.45). When dose groups were analyzed separately, treatment differences relative to placebo were not significant for 100 mg [0.22 (0.57); 95% CI for difference from placebo: -0.94, 1.38; *p* = 0.70] or for 300 mg [0.55 (0.59); 95% CI for difference from placebo: -0.65, 1.74; *p* = 0.36] (Fig. 3D).

### CSF biomarkers

**Fig. 4:**
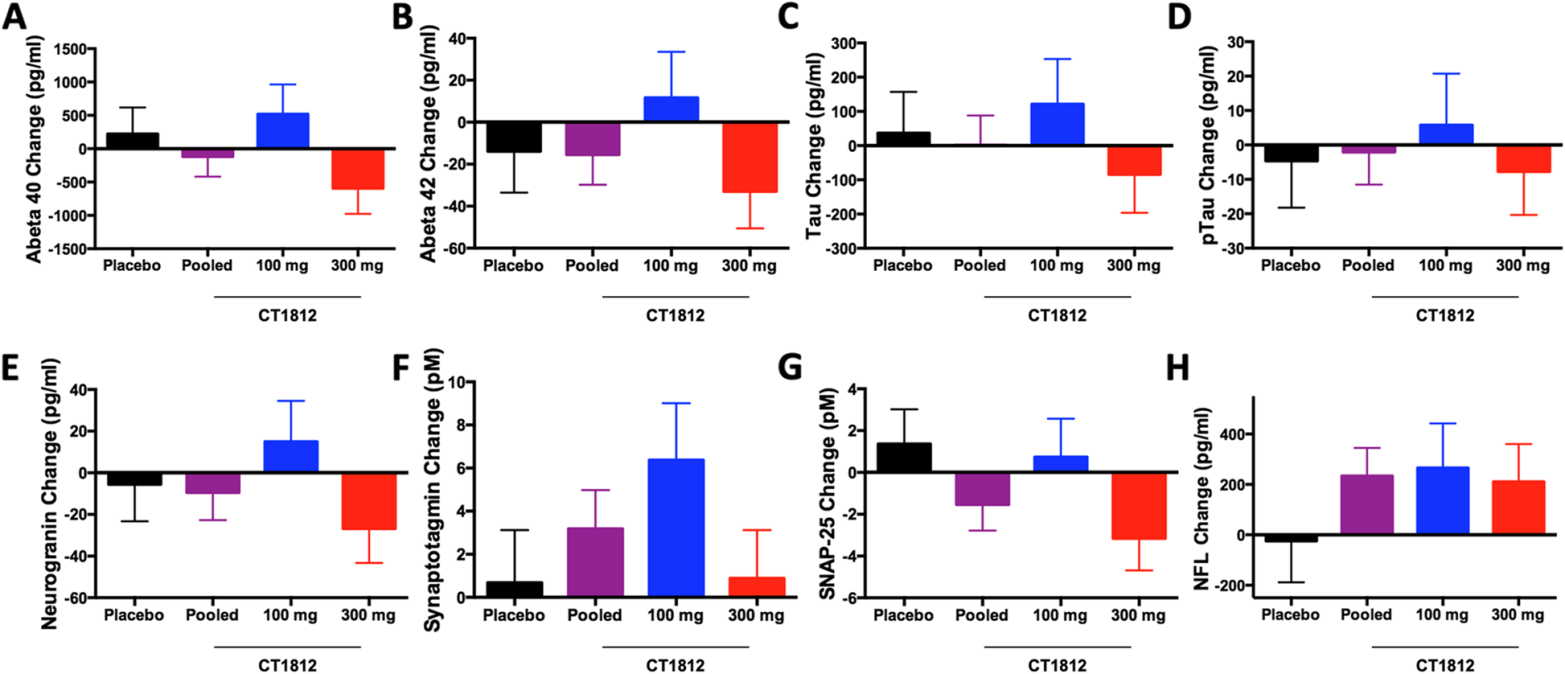
Change in CSF biomarkers from baseline at 24 weeks. CSF was collected at baseline and 24 weeks and analyzed for biomarkers associated with AD including Aβ40 (**A**), Aβ42 (**B**), Tau (**C**), pTau (**D**), neurogranin (**E**), synaptotagmin (**F**), SNAP-25 (**G**), and NfL (**H**). The mean changes from baseline (error bars: SE) are shown. No significant differences were demonstrated between any treatment groups in the change from baseline at 24 weeks

No dose- or treatment-related changes in CSF Aβ40, Aβ42, Tau, pTau, neurogranin, synaptotagmin, SNAP-25, or NfL were observed following 24 weeks of treatment with CT1812 (Fig. 4). The LS mean (SE) change from baseline at 24 weeks for these pharmacodynamic biomarkers of AD are listed in Supplemental Table 4. None of these differences were significant compared to placebo.

### Safety

The safety results for the primary 6-month study period are summarized in Table 2. No deaths occurred in the study. Five participants were withdrawn from the study due to SAEs/treatment-emergent AEs (TEAEs). A total of five SAEs occurred (in four participants with four occurring in the 6-month primary study period and one in the optional 6-month extension period): psychotic disorder (moderate), seizure (severe), thalamic infarct (severe) encephalitis (severe), and ureterolithiasis (moderate, occurred in the extension period), all deemed unrelated or unlikely related to study treatment. Four SAEs were in active study drug participants; the encephalitis event occurred in a placebo participant. Two participants in the 300 mg per day dose group were withdrawn from the primary study due to TEAEs of liver function test increases (mild, probably related to study treatment); in both cases, the AE was considered resolved on the day of withdrawal, there were no associated bilirubin abnormalities, and the participants were asymptomatic. TEAEs were reported for 18 of 23 participants (78%) across the three treatment groups, for a total of 52 TEAEs. In this period, the most common TEAE in the CT1812 treatment arms (reported in at least 3/16 participants who received CT1812) was headache, reported in 5 total participants who received CT1812 (2 of 8 subjects [25%] receiving 100 mg and 3 of 8 participants [38%] receiving 300 mg CT1812) and also in 1 of 8 participants (14%) receiving placebo. Thirteen TEAEs were deemed related (probably or possibly) to study treatment in a total of 10 of 23 participants (43%). Most related TEAEs were considered mild in severity (12 of 13 TEAEs). One TEAE was of moderate severity (dizziness, in the 300 mg treatment group, deemed possibly related).

Two participants (both in the CT1812 100 mg treatment group) were found to have post-dose neurological examination findings assessed as clinically significant, one related to coordination (bilateral ideomotor apraxia) and one related to mental state (presence of delusions), both deemed unrelated to study drug. Two CT1812-treated participants developed transient suicidal ideation as assessed by the C-SSRS. In both participants the suicidal ideation occurred at only a single clinical visit with no recurrences. No other clear safety differences were observed between CT1812 or placebo treatment as demonstrated by clinical laboratory tests, vital signs, ECG assessments, or physical or neurological examination findings, and no dose-related trends were observed among participants who received CT1812. All study MRI scans were read clinically by a neuroradiologist and revealed no significant changes, including treatment-emergent amyloid related imaging abnormalities (ARIA).

Safety findings within the thirteen subjects who entered the optional six-month extension period were essentially the same as in the primary study period and are summarized in supplemental Table 5. In the extension period there were no deaths, no additional discontinuations due to AEs, and no SAEs attributed to study drug. There was one additional treatment-related TEAE (headache, in placebo group) and one additional SAE (ureterolithiasis, in 100 mg group).

## Discussion

This phase 1b randomized clinical trial demonstrated that CT1812 at doses of 100 or 300 mg was reasonably safe and well tolerated in participants with mild to moderate dementia due to AD with no clear safety differences between CT1812 or placebo treatment groups. In comparison to placebo, CT1812 treatment in the combined active treatment groups had no significant effect on the change from baseline at 24 weeks in [^11^C]UCB-J PET-measured SV2A binding as a measure of synaptic density, or in [^18^F]FDG PET-measured reduction in glucose metabolism in an AD-associated composite region of interest. Interestingly, volumetric MRI analyses suggested a trend (*p* = 0.06) toward tissue preservation in a composite brain region in participants treated with CT1812. In an exploratory analysis of 16 regions, significant changes (*p* ≤0.05) compared to placebo were found in the pericentral cortex, prefrontal cortex, and hippocampus. While the sample size of the present study is small and there were no treatment differences on the cognitive clinical rating scales or in the CSF biomarkers, changes in ADAS-Cog in the placebo treated group were similar to historical and more recently reported changes from other studies [38–43].

### Safety and tolerability

In this study, the most common TEAEs were headache and dizziness. In all completed studies in AD patients taking CT1812, including this study (NCT02907567, NCT03522129 and NCT03493282) there were no treatment-related SAEs or deaths and the most common TEAEs reported were headache, lymphopenia, upper respiratory tract infection, cough, dizziness, liver function test increase, nasopharyngitis, and urinary tract infection. While CT1812 was reasonably safe and well tolerated in patients with mild to moderate dementia due to AD, of note, elevation of liver enzymes that return to normal levels following discontinuation of the drug have been observed in this and other studies. These elevations have not been associated with changes in bilirubin.

### Absence of a treatment effect on synapse density

CT1812 has been shown to restore synaptic protein expression and synapse number in vitro [8]. Aβ oligomers induce reversible spine retraction in vitro [44] with a corresponding loss of synapses and synaptic proteins [4, 9]. The effect of CT1812-induced displacement of Aβ oligomers on oligomer-induced spine and synapse loss in cultured rat neurons has been investigated using the cytoskeleton binding protein drebrin as a marker of synapse number [8]. The addition of Aβ oligomers to neuronal cultures induced synaptic loss of a magnitude similar to that observed using ultrastructural stereology methods in postmortem hippocampus from humans diagnosed with MCI [45]. Moreover, the addition of CT1812 to cultures one hour after the addition of Aβ oligomers resulted in a concentration-dependent increase of synaptic number to normal levels [8]. Finally, examination of the expression of the synaptic proteins neurogranin and synaptotagmin-1 corroborated these results. Treatment with CT1812 blocked this loss and restored expression of both proteins to control levels. This suggests that CT1812-mediated displacement of Aβ oligomers from neuronal synapses stops both downstream oligomer-induced synaptic protein downregulation and synapse loss, facilitating synaptic recovery from toxic oligomer insult.

The present study was designed and powered to detect a rapid restoration of synapses similar to that observed in cultured cells with CT1812 [8] or in transgenic mouse models as has been demonstrated with other therapeutic agents [46–49]. We should note that synapse restoration to our knowledge has never been demonstrated in patients with AD with therapeutic interventions, although the technology to measure it in vivo with SV2A PET is a relatively recent development. Another therapeutic agent that demonstrated rapid restoration of synapses in transgenic mice [48] failed to show rapid restoration of [^18^F]FDG metabolism as a correlate of synaptic activity in a 4-week Phase 1b study [50] or slowing of decline in [^18^F]FDG metabolism or clinical outcomes in a 12-month Phase 2a proof of concept study [51]. No therapeutic agent has yet been tested with synaptic density PET in a fully-powered trial of longer duration. Whether the synapse restoration seen in preclinical studies will translate to human AD therapeutic trials remains to be seen. Perhaps only a slowing of synaptic loss is a feasible aim in human AD trials. The testing of that aim will require the incorporation of synaptic PET imaging in trials of longer duration that are fully-powered to detect a slowing of synaptic density loss. Furthermore, additional longitudinal observational experience with SV2A PET is needed as a prelude to such intervention trials.

### Effect of CT1812 on MRI volumes

This 24-week clinical trial demonstrated a trend toward slowing the rate of brain volume loss on the volumetric MRI regional composite. Moreover, in an exploratory analysis of 16 different brain regions, treatment with CT1812 showed a significant slowing in the rate of volume loss compared to placebo-treated participants in the hippocampus and in the prefrontal and pericentral cortices. Brain volume loss has been associated with pathogenetic changes (e.g., the accumulation of amyloid plaques and neurofibrillary tangles), as well as specific neurodegenerative changes (e.g., neuronal and synaptic loss) and cognitive deterioration in AD patients [52–54]. Although the composite region measured in this study is not directly comparable to other studies, the relative preservation of brain volume associated with CT1812 treatment compared to placebo in this study may suggest potential beneficial effects of CT1812 on some of these processes. A Phase 2 proof-of-concept study assessing the effect of CT1812 over 18 months (ClinicalTrials.gov Identifier: NCT05531656) will therefore incorporate brain volume measurements as a secondary outcome measure.

### Limitations

The major limitations of this trial, as previously noted, were the small sample and short treatment duration, which were sufficient to detect only a rapid and large restoration of synapses. It is possible that potential benefits of CT1812 could be observed in larger and longer studies. In addition, the power to detect longitudinal changes in brain volume may be improved in future Phase II studies by using an MRI analysis method that creates an unbiased within-subject template space, such as the FreeSurfer Longitudinal Processing Pipeline [55].

## Conclusion

Overall, CT1812 was generally well tolerated when delivered daily over a 24-week period in subjects with mild to moderate dementia due to AD. While no significant differences from placebo were seen in cognitive outcomes, [^11^C]UCB-J PET or [^18^F]FDG PET, or CSF biomarker outcomes, volumetric MRI findings suggested a trend towards brain tissue preservation following CT1812 treatment. These findings support the continued development of CT1812 for the treatment of patients with AD.

### Supplementary Information

**Additional file 1.** Statistical Analysis Plan.

**Additional file 2: Supplemental Table 1.** Composite ROI of AD-affected brain regions. **Supplemental Table 2.** Exploratory brain regions. **Supplemental Table 3.** Volumetric MRI. **Supplemental Table 4.** CSF pharmacodynamic biomarkers. **Supplemental Table 5.** Safety including optional 6 month extension period (total 12 months).

## Data Availability

All data needed to evaluate the conclusions in the paper are presented in the paper itself, in the supplementary materials, or in additional data that are available upon request from the authors.

## Abbreviations

HRRT: High-resolution research tomograph
MPRAGE: Sag 3D magnetization-prepared rapid gradient-echo
