# Supplementary material for: A pilot study to evaluate the effect of CT1812 treatment on synaptic density and other biomarkers in Alzheimer’s disease
Source: Alzheimers Res Ther. 2024 Jan 25;16:20. doi: 10.1186/s13195-024-01382-2 (PMC10809445; doi:10.1186/s13195-024-01382-2)
Supplement: Supplementary file 1 — Additional file 1. Statistical Analysis Plan. [file 13195_2024_1382_MOESM1_ESM.pdf]

Statistical Analysis Plan – COG0105

TITLE: A PILOT SYNAPTIC VESICLE GLYCOPROTEIN 2A (SV2A) PET STUDY TO EVALUATE THE EFFECT OF CT1812 TREATMENT ON SYNAPTIC DENSITY IN PARTICIPANTS WITH MILD TO MODERATE ALZHEIMER'S DISEASE

STUDY SPONSOR:

Cognition Therapeutics, Inc.
2403 Sidney St., Suite 261
Pittsburgh, PA 15203
USA

SPONSOR CONTACT:

Susan Catalano, Ph.D.
Chief Science Officer
Cognition Therapeutics, Inc.

Email: scatalano@cogrx.com

Document History:

**Document**	**Version**	**Version Date**
Statistical Analysis Plan – COG0105	Final 1.0	11 Mar 2021

CONFIDENTIALITY STATEMENT:

All information concerning the study drug supplied by the Sponsor in connection with this study, and not previously published, is considered confidential and proprietary information. This information includes the Investigator's Brochure, clinical protocol, subject information and informed consent, and case report forms. This confidential information shall remain the sole property of the Sponsor, shall not be disclosed to others without prior written consent from the Sponsor and shall not be used except in the performance of this study.

The information developed during the conduct of this clinical study is also considered confidential and will be used by the Sponsor in connection with the development of the study drug. This information may be disclosed as deemed necessary by the Sponsor.

This Statistical Analysis Plan has been prepared by Avance Clinical Pty Ltd (Avance) for the COG0105 study (Cognition Therapeutics, Inc.). The contents of this procedure are confidential and reproduction, in part or whole, is not permitted without prior written permission from Avance and the Sponsor.

DocuSigned by:

Signer Name: Rene Kroon
Signing Reason: I am the author of this document
Signing Time: 11-Mar-2021 | 15:19:08 ACDT
BFCD598BC92B4F0494B97845786FF6E0

11-Mar-2021 | 15:19:41 ACDT

Prepared by: Date
René Kroon
Biometrics Team Manager / Senior Biostatistician
Avance Clinical Pty Ltd

DocuSigned by:
Bilyana Dabin
Signer Name: Bilyana Dabin
Signing Reason: I have reviewed this document
Signing Time: 11-Mar-2021 | 15:29:24 ACDT
F9AB6AA112CB4BDCA1381170F727B78A

11-Mar-2021 | 15:29:35 ACDT

Reviewed by: Date
Bilyana Dabin
Principal Biostatistician / Pharmacokineticist
Avance Clinical Pty Ltd

DocuSigned by:
Michael Grundman
Signer Name: Michael Grundman
Signing Reason: I approve this document
Signing Time: 11-Mar-2021 | 09:57:27 PST
7B770A14B0D84DCBB4CAEA5E798A6AAA

11-Mar-2021 | 09:57:38 PST

Approved by: Date
Michael Grundman, M.D.
Senior Clinical Advisor
Cognition Therapeutics, Inc.

TABLE OF CONTENTS

1. Scope of the Statistical Analysis Plan ... 6
2. Abbreviations ... 6
3. Relevant Avance Standard Operating Procedures and Guidances ... 7
4. Introduction ... 7
 4.1. Study Overview ... 7
 4.2. Study Objectives ... 7
 4.3. Study Design ... 8
 4.4. Study Treatment ... 8
 4.5. Sample Size ... 8
 4.6. Study Endpoints ... 9
5. Data Listings ... 10
 5.1. Sources of Data ... 10
 5.2. Randomization and Subject Identification Code ... 11
 5.3. Maintaining the Study Blind ... 11
 5.4. Assessment Time Point Identifiers ... 11
 5.5. Data Derived by Calculation ... 11
 5.6. Handling of Missing Data ... 15
 5.7. Coding Abbreviations ... 17
6. Analysis Populations ... 17
 6.1. Safety Analysis Set ... 17
 6.2. Efficacy Analysis Set ... 18
 6.3. Pharmacokinetics Analysis Set ... 18
 6.4. Pharmacodynamic Analysis Set ... 18
7. Subject Disposition/Background Data ... 19
 7.1. Subject Disposition ... 19
 7.2. Baseline and Eligibility Assessments ... 19
 7.3. Baseline Data Analysis ... 19
8. Safety Data ... 20
 8.1. Safety Assessments ... 20
 8.2. Safety Data Presentation and Analysis ... 20
9. Efficacy Data ... 21
 9.1. Efficacy Assessments ... 21
 9.2. Efficacy Data Presentation ... 22
 9.3. Efficacy Analysis ... 22
 9.4. Other Exploratory Efficacy Analyses ... 23
 9.5. Adjustment for Multiplicity ... 25
10. imaging outcomes ... 25
 10.1. [^{11}C] UCB-J PET Distribution Volume Ratio (DVR) ... 25
 10.2. [^{18}F]FDG PET SUV ratio (SUVR) ... 26
 10.3. Volumetric MRI ... 26
 10.4. Intrinsic Connectivity Contrast (ICC) ... 27
11. Pharmacokinetic/Pharmacodynamic Data ... 28
 11.1. Pharmacokinetic Sample Collection ... 28
 11.2. Pharmacokinetic Concentrations ... 28
 11.3. Pharmacokinetic Parameters ... 29

11.4. Statistical Analysis of Pharmacokinetic Parameters 29
11.5. Pharmacodynamic Endpoints 29
11.6. Pharmacodynamic Analysis 30
11.7. Statistical Analysis of Pharmacodynamic Parameters 30
11.8. Changes from the Analysis Planned in the Protocol 30
12. Data Listings and Summary Tables 30
12.1. Interim/Preliminary Data Analysis 30
12.2. Listings and Tables for Clinical Study Report 30
12.3. Data Transfer to the Study Sponsor 30
13. General Considerations for Data Management and Analysis 31
13.1. Analysis Packages 31
13.2. Electronic Data Management 31
13.3. Archiving 31

APPENDICES

Appendix 1: Planned Data Listings 32
Appendix 2: Planned Summary Tables 34

1. SCOPE OF THE STATISTICAL ANALYSIS PLAN

This Statistical Analysis Plan is an adjunct to Cognition Therapeutics, Inc. Protocol No. COG0105 dated 03 February 2021, Version 1.5. The Statistical Analysis Plan details the pre-planned procedures for the pharmacokinetic and statistical methods used in the analysis of the clinical data.

Additional analyses may be explored as well upon review of the unblinded results and will be considered as post hoc analyses.

2. ABBREVIATIONS

AD	Alzheimer's disease
ADAS-Cog	Alzheimer's Disease Assessment Scale – cognition subscale
ADCS-ADL	Alzheimer's Disease Cooperative Study – Activities of Daily Living
ADCS-CGIC	Alzheimer's Disease Cooperative Study – Clinical Global Impression of Change
AE	Adverse event
ANCOVA	Analysis of covariance
BMI	Body mass index
CDR-SB	Clinical Dementia Rating Scale Sum of Boxes
CRF	Case report form
DVR	Distribution volume ratio
ECG	Electrocardiogram
fMRI	functional magnetic resonance imaging
GDS	Geriatric Depression Scale
ICC	Intrinsic Connectivity Contrast
ICH	International Council for Harmonisation of Technical Requirements for Pharmaceuticals for Human Use
IP	Investigational Product
MedDRA	Medical Dictionary for Regulatory Activities
MMSE	Mini Mental State Exam
NTB	Neuropsychological Test Battery
PD	Pharmacodynamic
PK	Pharmacokinetic
QA	Quality assurance
QC	Quality control
SAE	Serious adverse event

SAP	Statistical analysis plan
SMC	Safety monitoring committee
SOP	Standard operating procedure
SUVR	SUV ratio
WHODrug	WHO Drug Dictionary

3. RELEVANT AVANCE STANDARD OPERATING PROCEDURES AND GUIDANCES

The following Avance SOPs and regulatory guidances are relevant to this Statistical Analysis Plan:

- SOP BIOM-0002 Clinical Data Analysis and Presentation
- SOP BIOM-0003 Blinding: Codes and Code Breaking
- SOP BIOM-0004 Randomisation Generation
- SOP BIOM-0007 Pharmacokinetic Analysis Using WinNonlin
- SOP BIOM-0009 Statistical Analysis Plans
- ICH: Statistical Principles for Clinical Trials (ICH E9, Current Step 4 version dated 5 February 1998)
- FDA: Bioavailability and Bioequivalence Studies for Orally Administered Drug Products – General Considerations (Revision 1, March 2003).
- Bioavailability Studies Submitted in NDAs or INDs – General Considerations (latest draft February 2019)

4. INTRODUCTION

4.1. Study Overview

This is a randomized, double-blind, placebo-controlled, parallel-group trial evaluating CT1812 with planned dose levels of 100 mg and 300 mg.

4.2. Study Objectives

Primary:

- To evaluate the safety and tolerability of CT1812 in AD patients

Secondary:

- To evaluate the effect of CT1812 on brain synaptic density in AD patients using the SV2A PET ligand ^{11}C-UCB-J.
- To evaluate the effect of CT1812 on cognitive and clinical outcomes in AD patients using the ADAS-COG, NTB, derived cognitive composites, ADCS-ADL, MMSE, CDR-SB, and ADCS-CGIC

- To evaluate the effect of CT1812 on brain activity in AD patients using FDG PET, resting state functional MRI
- To evaluate the effect of CT1812 on brain volume in AD patients using volumetric MRI
- To evaluate the effect of CT1812 on CSF pharmacodynamics in AD patients through measurement of CSF Aβ 40, Aβ 42, tau, phospho-tau, neurogranin, synpatosomal-associated protein-25 (SNAP25), synapotagmin, and NFL. Additional CSF biomarkers are listed in Section 11.5.
- To evaluate the plasma and CSF concentrations of CT1812 in AD patients.

Exploratory:

- To correlate changes in synaptic density, other imaging endpoints and biomarkers, with cognitive function following CT1812 treatment.
- To evaluate the plasma CT1812 metabolites in AD patients

4.3. Study Design

This is a single-center, Phase 1b, randomized, double-blind, placebo-controlled, parallel-group trial in adults with mild to moderate AD.

After consenting to participate in the study, screening procedures will occur between Days -60 and -1. Eligible participants will be randomized in a 1:1:1 (300 mg active: 100 mg active: placebo) ratio. The first dose of study drug will be administered in the clinic after all baseline procedures have been conducted. For the remainder of study days, participants will ingest study drug each morning at home. Participants and their study partners will return to the clinic for repeat psychometric/neurologic testing, safety studies, and PK and PD sample collection approximately every 2 weeks for the first 6 weeks and then every 3 weeks thereafter up to 24 weeks for the primary study followed by an optional double-blind extension treatment period of an additional 24 weeks (337 days +/-2) as well as a follow up visit at 2 weeks after the final treatment visit

Participants who prematurely discontinue the study for any reason will be asked to attend a final safety and efficacy visit.

4.4. Study Treatment

CT1812 will be provided as a hydroxypropyl methylcellulose (HPMC) capsule containing 191 mg or 64 mg of CT1812 fumarate salt (equivalent to 150 mg or 50 mg of the CT1812 free base, respectively).

Placebo capsules (containing of 191 mg or 64 mg of lactose monohydrate) will be supplied to match the CT1812 supplies in the same packaging.

CT1812 or matching placebo will be administered once each morning as a 300 mg or 100 mg oral dose (2 capsules).

4.5. Sample Size

Computation of sample size in this within-participant design was based on estimates of the magnitude of reductions in synaptic density from post-mortem AD data of 25-45%. It

was assumed that, on average, there is 30% loss of synapses in this population, and that CT1812 would restore one third of those synapses, with no change in synaptic density in the placebo group. Based on the excellent test/retest reliability of ^{11}C UCB J, it was conservatively assumed 10% variability in baseline and post-treatment ^{11}C UCB J binding potential non-displaceable (BPND) measurements. Power was first computed by assessing whether the change in synaptic density would be larger in the treated group than the placebo group.

For group sizes of 7 per group, one has ~68% power for comparison of the combined treatment of N=14 CT1812 vs. 7 placebo subjects (α = 0.05, 1-tailed test). Higher power was present in the one-sample test of the treatment group, i.e., did synaptic density increase following CT1812 treatment. In that case, a group size of 7 CT1812 patients yields 80% power to detect an increase in synaptic density. Power for detecting changes in 18F FDG is likely to be lower since the magnitude of change in the SV2A signal in AD will be larger than that of 18F FDG (20%).

4.6. Study Endpoints

Safety Endpoints:

- The incidence and severity of adverse events (AE)
- The incidence of serious adverse events (SAE)
- Changes in vital signs
- Changes in physical and neurological exam findings
- Changes in body mass
- Changes in electrocardiogram (ECG) findings
- Changes in clinical laboratory testing (serum chemistry, hematology, urinalysis)
- Changes in the Columbia Suicide Severity Rating Scale (C-SSRS)

Secondary:

- The primary imaging endpoint is the change from the baseline in synaptic density as measured by differences in SV2A PET ligand ^{11}C-UCB-J uptake after dosing with CT1812 versus placebo.
- Cognitive and clinical outcomes:
  - Mini Mental State Exam (MMSE).
  - Alzheimer's Disease Assessment Scale-Cognitive Subscale (ADAS-Cog) 11 and ADAS-Cog13 (delayed recall and Number Cancellation added to ADAS-Cog11 in the ADAS-Cog13); ADAS-Cog14 includes the maze added to ADAS-Cog13.
  - Neuropsychological Test Battery (NTB) including Trails A and B, Digit Span, and Letter and Category Fluency (CFT).
  - Alzheimer's Disease Cooperative Study (ADCS)-Clinical Global Impression of Change (CGIC).

  - ADCS-Activities of Daily Living (ADCS-ADL).
  - Clinical Dementia Rating Scale – Sum of Boxes (CDR-SB)
  - Composite z-score scales (Cognitive Composite score; Memory composite score; Attention composite score; Executive Function composite score).
  - CSF pharmacodynamics in AD patients through measurement of CSF Aβ 40, Aβ 42, tau, phospho-tau, neurogranin, synaptotagmin, SNAP25, and NFL.
- Imaging Outcomes
  - FDG PET
  - Functional MRI
  - Volumetric MRI

Pharmacokinetic Endpoints:

- The following CT1812 pharmacokinetic assessments will be made based on serial pre-dose concentrations in plasma and CSF:
  - CT1812 CSF/plasma concentration ratio
  - Changes in pre-dose CT1812 plasma concentrations
  - Plasma CT1812 metabolites

5. DATA LISTINGS

5.1. Sources of Data

Data sets containing raw data will be provided by the Data Management group, extracted from the clinical study database of data entered into a Case Report Form. It is assumed that the database will contain the data from Screening through to the Follow-up Safety Visit of the study.

Analysis datasets will be generated from the data extracted from the study database, along with the following data entered separately or received from other sources:

- Randomisation/treatment assignment data, to be provided by the Avance unblinded statisticians
- Clinical laboratory safety data, to be provided by Quest Diagnostics to Avance Clinical data management
- CSF and plasma PK concentration data, to be provided by Agilex Biolabs
- PD concentration data (CSF) data, to be provided by Agilex Biolabs

All data entered into the CRF or provided from other sources as described above will be presented (explicitly or implicitly) in data listings or figures as described in Appendix 1. All available data collected for screen failed individuals will also be included in the individual data listings.

5.2. Randomization and Subject Identification Code

This is a double-blind, placebo-controlled study. Study drug will consist of either of two doses of CT1812 or placebo. The placebo will be identical in appearance to both doses of the active CT1812.

The non-blinded statistician assigned to the trial will generate a list with the appropriate number of 4 digit individual study IDs randomly for each arm, randomly assigned to either CT1812 300 mg, CT1812 100 mg or placebo treatment in a 1:1:1 ratio. Randomization will include a stratification factor for the MMSE score (18-22 or 23-26).

The Randomisation Number will be specified as Rrnnn, where:

- nnn = sequential subject id from 001 to 999,

In the listings presenting the individual data, subjects will be identified by randomization number, with dose of CT1812 as a secondary grouping identifier. The screening number will be shown only on the listing of informed consent data.

5.3. Maintaining the Study Blind

In this double-blind study, all personnel involved, i.e. physicians, site staff, and participants will remain blinded at all times, except in an emergency where knowledge of the randomization code is required to provide appropriate treatment. The SMC will remain blinded but may break the blind for a given subject if deemed justified in accordance with the committee charter.

Preparation of the randomization schedule in accordance with Avance SOP S-DM-004 (Randomization) was performed by staff members of Avance who have no responsibility for monitoring and data management of this study.

The individual data listings for inclusion in an appendix to the report will be prepared after the study has been unblinded.

5.4. Assessment Time Point Identifiers

In the data listings for the report, scheduled assessment time points will be identified as follows:

- Scheduled safety assessments will be identified by treatment, study day, and also nominal study time point, where relevant.
- Pharmacokinetic assessments will be identified by nominal time post-dose for the study treatment period.
- Assessments at Screening and Study Completion visit will be identified as such for both study day and time-point, as appropriate.

5.5. Data Derived by Calculation

The following data fields for inclusion in the data listings will be derived by calculation:

AEs Single Period:

- Treatment-emergent Indicator for adverse event, assigned as
  Treatment-Emergent if

(Onset Date + Onset Time) >= (Date of First Dose + Time of First Dose)

Non Treatment-Emergent if
(Onset Date + Onset Time) < (Date of First Dose + Time of First Dose)

- Adverse event time since first dose (in days, to two decimal places), calculated as
  (Onset Date + Onset Time) – (Date of First Dose + Time of First Dose)
- Adverse event time since dose in current treatment period (in days, to two decimal places), calculated as
  (Onset Date + Onset Time – Date of Dose in Current Treatment Period - Time of Dose in Current Treatment Period)

AE Duration:

- Adverse event duration (in days, to two decimal places), calculated as
  (Resolution Date + Resolution Time – Onset Date - Onset Time)

Safety Assessments Change from Baseline:

- Baseline for Vital Signs, ECG parameters, serum clinical laboratory parameters will be the most recent scheduled assessment prior to first dose

Clinical Laboratory Assessments:

- If a clinical laboratory result is above the upper limit of normal, then Out of Range flag will be set to "H"
- If a clinical laboratory result is below the lower limit of normal, then Out of Range flag will be set to "L"
- Clinical Laboratory parameters that are outside the reference range and assessed as clinically significant will be identified as "Abnormal CS"
- Clinical Laboratory parameters that are outside the reference range and not assessed as clinically significant will be identified as "Abnormal NCS"

Pharmacokinetics:

- Actual collection time post-dose (in hours, to two decimal places) for PK blood samples for each dose day calculated as
  (Collection Date +Collection Time - Date of Dose - Time of Dose)

Pharmacodynamics:

- Actual collection time post-dose (in hours, to two decimal places) for PD CSF samples for each dose day calculated as
  (Collection Date +Collection Time - Date of Dose - Time of Dose)

Efficacy Assessments Change from Baseline:

- Baseline for ADAS-Cog11, ADAS-Cog13, ADAS-Cog14, NTB, ADCS-ADL, MMSE, CDR-SB, and ADCS-CGIC will be the most recent scheduled assessment prior to first dose

Efficacy Assessments:

- MMSE Total Score = sum of the individual scores from the 11 questions on the scale.
- The ADAS-Cog11 total score = the sum of all 11 individual items (word recall; commands; constructional praxis; naming objects and fingers; ideational praxis; orientation; word recognition; remembering test instructions; spoken language; word finding; and comprehension of spoken language). The ADAS-Cog 11 will be considered the primary cognitive endpoint for this study.
- The ADAS-Cog13 total score includes all of the items in the ADAS-Cog11 and the delayed word recall and the number cancellation.
- The ADAS-Cog14 total score includes all of the items in the ADAS-Cog13 and the maze item.
- The Cognitive Composite score will be calculated using z-scores of the below items. The z-scores will be derived by calculating the baseline mean and standard deviation of each individual component for all subjects. A z-score within each component will be derived for each subject at each timepoint by subtracting the corresponding baseline mean and then dividing by the corresponding baseline SD. The Cognitive Composite score will be set as the mean of the 10 individual z-scores within each subject and timepoint. If a subject is missing one, two, or three scores at a particular timepoint, the missing scores will be set as the average non-missing z-score of all subjects at that timepoint within each component when deriving the Cognitive Composite score. If a subject is missing four or more individual components at each timepoint, the Cognitive Composite score will not be derived.

  ADAS-COG

  Word recall (scored on a 0 to 30 range), sum of trials I, II, and III. If at least one of the trials is missing, this component will be considered missing. The sign of the z-score for this component will be reversed when deriving the Cognitive Composite score as it is the total words not recalled that is being recorded. This test is a key measure of episodic memory.

  Orientation (0 to 8). The sign of the z-score for this component will be reversed when deriving the Cognitive Composite score as the recorded score for this test is the number of items answered incorrectly. This measure is an index of the study participant's episodic memory and a single item of semantic memory (their name).

  Delayed Word Recall (0 to 10). The sign of the z-score for this component will be reversed when deriving the Cognitive Composite score as it is the total words not recalled that is being recorded.

  Word Recognition (0 to 24). The sign of the z-score for this component will be reversed when deriving the Cognitive Composite score. This is a further test of episodic memory using a word recognition paradigm of 12 true positive targets and 12 foil items. While the possible score for this measure is 0 to 24, the standard ADAS-Cog score is capped at a maximum worst score of 12.

Number Cancellation (0 to c.40). This test was added to the original ADAS-Cog to remedy the absence of an attentional test. Performance can range between a score of 0 and c.40. ADAS-Cog scoring requires that ‘False alarms’ be subtracted from ‘hits’ to yield a score which is reported as 0 to 5 for ADAS-Cog scoring purposes. Having ensured that the total number of false alarms are comparable across both arms of the study, we will analyze total hits. If false alarms are unbalanced across study arms then the statistic ‘D’ will be calculated using Signal Detection Theory.

Maze - The ADAS-Cog14 augments the ADAS-Cog11 with delayed free recall, number cancellation, and maze-completion measures. A score of 0 to 10 for delayed free recall and a conversion code of 0 to 5 for the digit cancellation and maze completion provides total score ranges for the extended ADAS-Cog14 from 0 to 90. The Maze item will be included as one of the components when deriving the Cognitive Composite and Attention Composite score. The sign of the z-score for this component will be reversed when deriving these composite scores.

NTB

CFT – This is a further test of working memory and executive function that yields a key metric referred to as ‘Total number of acceptable words’. This is obtained by calculating how many of the study participant’s responses are deemed acceptable according to the scoring rules. The usual performance range for this test is between 0 and 40.

Digit Span – This is a brief measure of working memory, especially in the Digits Backwards section. The key metric is ‘Total number of trials correct’, which is the number of sequences correctly recalled. The range of the test is 0 to 24 and both floor and ceiling effects are rare.

Trail Making Test (TMT) A – This is essentially a measure of attention, as successful completion relies on quickly and accurately connecting numbered circles in an ascending sequence. The key metric for analysis purposes is ‘Time taken for completion’. Errors and time taken once again both contribute to performance. However, because errors incur a time penalty, it is not necessary to analyze both metrics. The sign of the z-score for this component will be reversed when deriving the Cognitive Composite score.

TMTB – This is the more complex component of the TMT in which successful completion relies on the study participant accurately connecting the numbered and lettered circles. Study participants are required to alternate between numbers and letters in an ascending sequence. The key metric for analysis purposes is again ‘Time taken for completion’. As with TMT A, because errors incur a time penalty, it is not necessary to factor in errors for analysis purposes. The sign of the z-score for this component will be reversed when deriving the Cognitive Composite score.

- The Memory composite score will be a composite z-score average similar to the Cognitive Composite score, but will only be derived using the average of the

ADAS-Cog Word Recall, Orientation, Delayed Word Recall, and Word Recognition items. If a subject is missing any of the four items at a timepoint, this composite score will not be derived.

- The Attention composite score will be a composite z-score average derived using the average of the Number Cancellation, Maze item from ADAS-Cog 14, and TMT A items. If a subject is missing either of the three items at a timepoint, this composite score will not be derived.
- The Executive function composite score will be a composite z-score average derived using the average of the CFT, Digit Span, and TMT B items. If a subject is missing any of the three items at a timepoint, this composite score will not be derived.

5.6. Handling of Missing Data

The missing data for Cognitive composite is discussed in Section 5.5.

For adverse events, where either the onset time or resolution time is unknown, time since first dose and duration will be imputed as follows:

- If onset date is unknown, and it cannot be confirmed that onset was prior to the start of dose administration, then the AE will be classified as treatment-emergent, with unknown time since first dose.
- If either the date of onset or the date of resolution is unknown, then duration will be shown as unknown.
- If onset time is unknown:
    - If the date of onset is known to be the date of first dose administration, and it is confirmed by a CRF comment or communication with the site that onset was prior to the start of dose administration, then the AE will be classified as not treatment-emergent.
    - If the date of onset is known to be same as the date of first dose administration, and it is confirmed by a CRF comment or communication with the site that onset was after the start of dose administration, then Time since first dose will be shown on the listing as “<1” day.
    - If the date of onset is known to be later than the date of first dose administration, then Time since first dose will be determined as the number of days since first dose, shown on the listing as “~n” days, where n is the number of days difference between the date of dose administration and the date of onset.
- If either the onset time or resolution time is unknown:
    - If the date of onset and the date of resolution are known to be the same, then Duration will be shown on the listing as “<1” day.

- If the date of onset and the date of resolution are known and are different, then Duration will be determined as the number of days difference between the date of onset and the date of resolution, shown on the listing as “~n” days.

Baseline for safety assessments:

- Missing values for safety assessments will not be imputed. If change from baseline is to be determined and the pre-dose baseline assessment is missing, then the previous scheduled result (e.g. Screening or Day -1) will be used as the baseline. However, if the baseline is missing due to technical reasons and a subsequent unscheduled pre-dose assessment is used to confirm eligibility, this unscheduled assessment may be used as baseline.

Pharmacokinetic concentration and collection times:

- Missing PK concentrations will not be imputed, except:
  - If the Day 1 pre-dose concentration is missing and the analyte is not endogenous, then a value of zero will be imputed;

Efficacy missing data rules:

- The ADAS-Cog11 total score ranges from 0-70 with higher scores indicating greater dysfunction. When the total score is missing due to non-cognitive reasons for one of the questions, the total score will be calculated as a weighted average of the scores provided for the remaining ten questions as follows:
  - Imputed Total= Observed Total Score * (1+ Maximum Score of the missing value / Sum of the Maximum Score of the non - missing values)

Calculation of Imputed ADAS-Cog-11 Total Score

**Missing Question**	**Imputed Total formula**
Word Recall	Observed Total*(1+10/60)
Orientation	Observed total*(1+8/62)
Word Recognition	Observed total*(1+12/58)
All other questions	Observed total*(1+5/65)

In cases where 2 or more questions are missing, the total score will not be imputed and will be set to missing.

- The ADAS-Cog-13 total score ranges from 0-85. The imputed total score when there is only one item is missing due to non-cognitive reasons is presented below:

Calculation of Imputed ADAS-Cog-13 Total Score

Missing Question	Imputed Total formula
Word Recall	Observed Total*(1+10/75)
Orientation	Observed total*(1+8/77)
Word Recognition	Observed total*(1+12/73)
Delayed Word Recall	Observed total*(1+10/75)
All other questions	Observed total*(1+5/80)

- The ADAS-Cog-14 total score ranges from 0-90. The imputed total score when there is only one item is missing due to non-cognitive reasons is presented below:

Calculation of Imputed ADAS-Cog-14 Total Score

Missing Question	Imputed Total formula
Word Recall	Observed Total*(1+10/80)
Orientation	Observed total*(1+8/82)
Word Recognition	Observed total*(1+12/78)
Delayed Word Recall	Observed total*(1+10/80)
Maze	Observed total*(1+12/78)
All other questions	Observed total*(1+5/85)

- No further imputation for efficacy assessments. In order to have all subjects with any non-missing efficacy data contributing to the analysis, a mixed model for repeated measures (MMRM) will be used to take the missing data into account. Under the assumption that missing data are missing at random, inferences based on methods of MMRM estimation in mixed models are valid.

5.7. Coding Abbreviations

Any coding abbreviations used in data listings will be included in the key in a footnote, as appropriate.

6. ANALYSIS POPULATIONS

6.1. Safety Analysis Set

The safety analysis set will include all subjects who were administered with the investigational product (IP), including those who may have withdrawn prior to study completion.

Safety data will be presented for all subjects in the safety analysis set, and summarised by treatment group.

Subject disposition and background data, including demographics, relevant background, participation and compliance data, will be presented for all subjects in the safety analysis set.

Participants who are assigned a randomization number but withdraw prior to dosing will not be included in the safety analysis set. Available details of their participation and reason for withdrawal will be listed separately.

6.2. Efficacy Analysis Set

Efficacy data will be presented for all subjects who were administered with the IP, including those who may have withdrawn prior to study completion.

The full analysis set (FAS) for efficacy will include all subjects who receive IP and who have at least one post-dose assessment of any of the cognitive and clinical endpoints.

Summaries of efficacy assessments will be presented for the FAS analysis set, by treatment group.

6.3. Pharmacokinetics Analysis Set

Pharmacokinetic concentration data will be listed and presented graphically for all subjects who were administered with active IP, including those who may have withdrawn prior to study completion. Pre-dose trough plasma concentrations will be determined for all subjects IP in the pharmacokinetics analysis set, noting that this may be not applicable on a scheduled sampling day for subjects from whom PK sample collection was not completed.

The per protocol pharmacokinetic analysis set will include all subjects in the PK Analysis Set who have no major protocol violations that impact on pharmacokinetics. Subjects who do not complete the sampling per schedule, but pre-dose trough samples on at least one visit day (after Day 1), will be evaluated. Data from subjects who experience emesis on the day prior to pre-dose trough sample collection at, or before, two times the t_{max} observed in previous studies (2 hours post-dose) may be deleted from statistical analysis..

Summaries of concentration data will include only subjects in the per protocol pharmacokinetic analysis set. Pharmacokinetic concentration data will be summarised by treatment group.

6.4. Pharmacodynamic Analysis Set

Pharmacodynamic data will be presented for all subjects who were administered with IP, including those who may have withdrawn prior to study completion.

The per protocol pharmacodynamic analysis set will include all subjects who receive IP and who have at least one post-dose result for any CSF concentrations.

Summaries of pharmacodynamic assessments will be presented for the Pharmacodynamic analysis set, by treatment group.

7. SUBJECT DISPOSITION/BACKGROUND DATA

7.1. Subject Disposition

Subject disposition and administration procedures recorded in the CRF are as follows:

- Written informed consent
- Randomization
- Administration of IP
- Study completion /discontinuation
- Protocol deviations
- Additional comments

Details of participation and inclusion in analysis populations will be listed by subject. Completion status will be summarised by treatment group.

Details of administration of IP will be listed by subject. Exposure to IP will be summarised by treatment group.

Protocol deviations will be listed by subject. A summary of protocol deviations will be prepared for inclusion in the clinical study report.

7.2. Baseline and Eligibility Assessments

Baseline and eligibility assessments are as follows:

- Demographic details
- Medical, Surgical and Psychiatric history
- Physical examination
- Eligibility
- Serology (HIV, Hepatitis B and C)
- Pregnancy Tests
- Blood sampling for Apolipoprotein E (ApoE) genotyping
- Modified Hachinski exam
- Geriatric Depression Score (GDS)
- Alzheimer's Disease Diagnosis

7.3. Baseline Data Analysis

Demographics will be listed individually by subject and summarised by treatment group and overall.

Medical history and physical examination data at baseline and results of tests performed for eligibility will be listed by subject.

Baseline assessments of on-study measures will be listed and summarised along with post-dose assessments, as described below.

8. SAFETY DATA

8.1. Safety Assessments

Safety assessments are as follows:

- Adverse events and concomitant medications used:
  - Continuous monitoring throughout the study period
- Vital signs:
  - Body temperature, seated systolic and diastolic BP, pulse rate and respiratory rate
- Body mass
- Clinical laboratory tests:
  - Hematology - red blood cell count, erythrocyte mean corpuscular haemoglobin concentration (MCHC), erythrocyte mean corpuscular volume (MCV), hematocrit, hemoglobin, leukocyte count, and absolute counts of monocytes, neutrophils, basophils, eosinophils and platelets
  - Chemistry - glucose, calcium, albumin, total protein, sodium, potassium, bicarbonate, chloride, magnesium, BUN, creatinine, creatine kinase, alkaline phosphatase, ALT, AST, bilirubin, lipase, lactate dehydrogenase (LDH) and phosphorus
  - Coagulation –INR, prothrombin time.
  - Urinalysis – osmolality, creatinine, calcium, sodium, turbidity, color, specific gravity, pH, protein, glucose, ketones, bilirubin, blood, urobilinogen, nitrite, leukocytes, and microscopic particles
  - Thyroid Tests – TSH, T3, T4
- ECG (ventricular rate, PR, QRS, QT, QTcF (Fridericia's formula for correction), QTcB (Bazett's formula for correction))
- Physical examination including all major organ systems (exception: genitourinary and reproductive should be symptom-directed).
- Symptom Directed Neurological Examination
- Columbia Suicide Severity Rating Scale (C-SSRS)

8.2. Safety Data Presentation and Analysis

All clinical safety and tolerability data will be listed for each subject.

Safety data will be summarized using descriptive statistics. Where summaries include changes from baseline, the relevant baseline value will be determined as described in Section 5.5 above.

All treatment-emergent adverse events reported in this study will be listed using MedDRA terms, as coded per the Data Management Plan. The number of subjects reported with AEs and the number of AEs reported following administration of the IP will be summarised by treatment, grouped according to system organ class and preferred term, using descriptive statistics. The order for system organ class will be according to the Internationally Agreed Order, as per the guidance document MedDRA® *Data Retrieval and Presentation: Points to Consider*, specific to the MedDRA version used for this study. Summaries of AEs will also be presented by severity and by relationship to investigational product. In these summaries, subjects will be counted only once per MedDRA term, for the AE of highest severity or least favourable relationship. Summaries will also be presented of SAEs and of AEs leading to study withdrawal.

Clinical laboratory parameters for clinical chemistry, hematology, coagulation, urinalysis and thyroid tests will be listed and summarised by treatment group and study timepoint. Medical assessments of blood and urine parameters will be summarised by treatment group and study timepoint. A summary of changes from baseline will also be presented for serum clinical laboratory parameters. Individual subject profiles will be presented for any laboratory parameters with at least one post-dose value outside the laboratory's reference ranges and deemed clinically significant.

Vital signs will be listed and summarised by treatment and study timepoint. A summary of changes from pre-dose baseline will also be presented.

Body mass will be listed and summarised by treatment and study timepoint. In addition, a summary of changes from pre-dose baseline will be presented.

ECG parameters will be listed and summarised by treatment and study time point. A summary of changes from pre-dose baseline will also be presented. For QTcB the number of subjects with values greater than 450 (and 480, 500) msec or an increase from baseline of at least 30 (and 60) msec will also be presented, in accordance with ICH E14 (Note for guidance on the clinical evaluation of QT/QTc interval prolongation and proarrhythmic potential for non-antiarrhythmic drugs, CHMP/ICH/2/04).

Changes in physical examination findings over time will be listed by body system for each subject. It is noted that any untoward findings identified on physical examinations after the administration of the first dose of study medication will be captured as an adverse event if those findings meet the definition of an adverse event as defined in the protocol.

Changes in neurological examination findings over time will be listed by neurological system for each subject.

Changes in the Columbia Suicide Severity Rating Scale (C-SSRS) will be listed for each subject.

9. EFFICACY DATA

9.1. Efficacy Assessments

Efficacy is assessed by the cognitive and clinical outcomes ADAS-Cog, NTB, ADCS-ADL, MMSE, CDR-SB and ADCS-CGIC scales, measured at the following timepoints:

- V1 (Study Day -60 to -1)/Step 1 (MMSE only)
- Baseline V2 (Study Day 1) (excluding MMSE)
- V5 (Study Day 43)
- V7 (Study Day 85)
- V9 (Study Day 127)
- Final/Early Discontinuation V11 (Study Day 169) (for subjects participating in the Primary Study Only)
- V11 (Study Day 169) (for subjects participating in the Extension Study)
- V13 (Study Day 253) (for subjects participating in the Extension Study)
- Final/Early Discontinuation V15 (Study Day 337) (for subjects participating in the Extension Study)

9.2. Efficacy Data Presentation

All cognitive and clinical assessments for each subject will be presented in individual data listings.

For the ADAS-Cog (sub) scales, the total score for the corresponding items will be calculated as per Section 5.5. The total score and the Change from Baseline in the total score will be included in the individual data listings. Both the total score and the change from baseline will be summarized by visit and treatment group. Observed Mean change from Baseline (+/- SD) in ADAS-Cog total scores will be presented graphically by treatment group over time.

For NTB, the total time (in seconds) taken to complete both Trail A and Trail B will be presented in the same way as for the ADAS-Cog total score, as described above.

The total score for ADCS-ADL, for MMSE and for CDR-SB will be presented in the same way as for the ADAS-Cog total score, as described above.

The Composite z-score scales will be presented for each scale, in the same way as for the ADAS-Cog total score, as described above.

For ADCS-CGIC, the individual data listing will present the original score on the seven-point scale. In addition, the seven-point score will be collapsed to 3 groups, combining scores 1-3 to “Improved”, 4 to “No change”, and 5-7 to “Worsening”. This derived three-point scale will also be included in the individual data listing.

For the seven-point scale and the derived three-point scale, the number of subjects in each of the categories will be summarized by visit and treatment group using counts and percentages.

9.3. Efficacy Analysis

The change from baseline in the composite scores and clinical outcomes will be analyzed using a mixed effects model including fixed effects for treatment group (i.e. 300 mg active, 100 mg active, placebo), visit and treatment by visit interaction. In addition, the subject’s baseline score will be included as a covariate. For each time point up to and

including 6-months, the treatment differences between 300 mg active and placebo, and 100 mg active and placebo will be estimated from the model. In addition, the low and high CT1812 dosed groups will be pooled and compared with the placebo group. This analysis will be considered the primary analysis.

A separate pooled analysis and analysis by arm will also be performed for time points up to and including the 12-month time point.

Additionally, p-values and corresponding two-sided confidence intervals will be provided. The LS Mean Change from Baseline in ADAS-Cog total scores with 95% CI will be presented graphically by treatment group over time.

In the event that a scale or parameter only has a single post-baseline timepoint, an ANCOVA model will be run in place of the MMRM model without the visit and visit by treatment interaction terms. The ANCOVA model will include a fixed effect for treatment group, and the subject's outcome at baseline as a covariate. For parameters for which there are multiple post-baseline timepoints, ANCOVA models such as that just described above may also be run in addition to the MMRM model at various timepoints.

The change from baseline in the ADAS-Cog (sub) scale total score, total time (in seconds) taken to complete both Trail A and Trail B in the NTB, ADCS-ADL total score, MMSE, CDR-SB, and composite scores will be analysed using the MMRM model described above.

To compare 300 mg active vs placebo and 100 mg vs placebo statistically, observed values for ADCS-CGIC will be analyzed using the MMRM model described earlier without the baseline term. Additionally, frequencies and percentages in the ADCS-CGIC will be summarized by visit. Cochran-Mantel-Haenszel mean score statistics, using rank scores will be used to assess the difference in ADCS-CGIC responses at each visit between the CT1812 dose levels and placebo.

9.4. Other Exploratory Efficacy Analyses

Pearson correlation coefficients will be used to assess associations between imaging endpoints, CSF biomarkers and cognitive and behavioral changes (ADAS-11, ADAS-13, ADAS-14, Cognitive Composite, Memory Composite, Executive Function Composite, Attention Composite, NTB individual items, MMSE, CDR-SB, ADCS-ADL, and ADCS-CGIC). Correlations will be calculated for baseline imaging and biomarker values and baseline efficacy measures. Additionally, correlations between change from baseline in by-visit imaging and biomarker values and change from baseline in efficacy scales will be calculated.

The composite imaging parameters that will be used for the correlations with the cognitive and functional endpoints include: the DVR composite region, the FDG composite region, the volumetric MRI composite region and the ICC composite region. Additionally, for volumetric MRI the cerebral cortex region and the lateral ventricles will also be included. For all imaging modalities the following individual parameters will also be included:

- Hippocampus

- Entorhinal cortex
- Parahippocampal corte
- Amygdala
- Fusiform gyrus
- Lingual gyrus
- Inferior/middle temporal cortex
- Anterior cingulum
- Posterior cingulum
- Precuneus
- Prefrontal cortex
- Superior temporal cortex
- Lateral parietal cortex
- Lateral occipital cortex
- Pericentral cortex
- Medial occipital cortex

The CSF parameters that will be used for the correlations with the cognitive and functional endpoints include:

- Aβ 40 monomer
- Aβ 42 monomer
- Tau
- phospho-tau
- neurogranin
- Synaptotagmin
- synpatosomal-associated protein-25 (SNAP25)
- NFL
- YKL-40 ELISA

Additional CSF exploratory biomarkers that will be used for the correlations of the cognitive and functional endpoints include:

- neuronal pentraxin-1
- neuronal pentraxin -2
- neuronal pentraxin receptor
- complexin-2
- syntaxin-1b
- sytaxin-7
- gamma-synuclein
- rab GDI alpha
- Phosphatidylethanolamine-binding protein 1
- AP-2 complex subunit beta
- 14-3-3 protein zeta/delta

Additional exploratory plasma biomarkers and CSF biomarkers such as CSF Aβ oligomers and other phospho-tau species may be analyzed later when these results become available and may be included as a supplementary appendix to the CSR.

CT1812 concentrations in CSF and plasma will also be correlated with changes in biomarkers and cognitive measures.

Additional analyses and correlations may be explored as well (e.g., possibly including age, gender, education and ApoE) and will be discussed in the clinical study report (CSR).

9.5. Adjustment for Multiplicity

No adjustments for multiplicity will be made.

10. IMAGING OUTCOMES

The change from baseline in the outcome will be compared between the treatment groups using the MMRM or ANCOVA model described in section 9.3.

The pooled analysis and analysis by arm will be performed with data for the composite regions and for the individual exploratory regions collected for points up to and including the 6-month time point. This analysis will be considered the primary analysis.

A separate pooled analysis and analysis by arm will also be performed with data for the composite regions and for the individual exploratory regions at time points up to and including the 12-month time point.

10.1. [^{11}C] UCB-J PET Distribution Volume Ratio (DVR)

For ^{11}C-UCB-J, the primary imaging outcome measure is *DVR* as produced by the Simplified Reference Tissue Model (SRTM2) using dynamic scan data from 0 to 60 min and the whole cerebellum as a reference region. For *DVR*, a composite region will be determined, including: prefrontal, lateral temporal, posterior cingulate/precuneus, anterior cingulate, lateral parietal, medial temporal, and lateral occipital regions. For the ^{11}C-UCB-J DVR outcome composite region the primary model will be analyzed without reference to age and ApoE4 status, however additional models will be run that include age and ApoE4 status as sensitivity analyses. Analyses will also be conducted to assess the relationship of age and ApoE4 status with the dependent DVR variable and to determine if there is any imbalance on these variables between the treatment groups.

Regional analyses will first focus on the hippocampus since the recent cross-sectional study has shown this to be the region of largest group differences in synaptic density when comparing a group of participants with AD to healthy participants.

Individual exploratory regions include:

- Hippocampus
- Entorhinal cortex
- Parahippocampal cortex
- Amygdala
- Fusiform gyrus

- Lingual gyrus
- Inferior/middle temporal cortex
- Anterior cingulum
- Posterior cingulum
- Precuneus
- Prefrontal cortex
- Superior temporal cortex
- Lateral parietal cortex
- Lateral occipital cortex
- Pericentral cortex
- Medial occipital cortex

10.2. [^{18}F]FDG PET SUV ratio (SUVR)

For ^{18}F-FDG, the primary imaging outcome measure is the *SUVR* from 60-90 min post injection using whole cerebellum as a reference region. For *SUVR*, a composite region will be determined, including: prefrontal, lateral temporal, posterior cingulate/precuneus, anterior cingulate, lateral parietal, medial temporal, and lateral occipital regions.

Individual exploratory regions include:

- Hippocampus
- Entorhinal cortex
- Parahippocampal cortex
- Amygdala
- Fusiform gyrus
- Lingual gyrus
- Inferior/middle temporal cortex
- Anterior cingulum
- Posterior cingulum
- Precuneus
- Prefrontal cortex
- Superior temporal cortex
- Lateral parietal cortex
- Lateral occipital cortex
- Pericentral cortex
- Medial occipital cortex

10.3. Volumetric MRI

A composite region of AD affected brain regions will be determined, including: prefrontal, lateral temporal, posterior cingulate/precuneus, anterior cingulate, lateral parietal, medial temporal, and lateral occipital regions. An additional cerebral cortex region will also be analyzed.

Individual exploratory regions include:

- Hippocampus

- Entorhinal cortex
- Parahippocampal corte
- Amygdala
- Fusiform gyrus
- Lingual gyrus
- Inferior/middle temporal cortex
- Anterior cingulu
- Posterior cingulum
- Precuneus
- Prefrontal cortex
- Superior temporal cortex
- Lateral parietal cortex
- Lateral occipital cortex
- Pericentral cortex
- Medial occipital cortex
- Lateral ventricles

10.4. Intrinsic Connectivity Contrast (ICC)

For resting state functional MRI, the outcome will be ICC. With this approach a map of the total connectivity of each voxel to all other voxels is computed. For ICC, a composite region of AD affected brain regions will be determined, including: prefrontal, lateral temporal, posterior cingulate/precuneus, anterior cingulate, lateral parietal, medial temporal, and lateral occipital regions.

Individual exploratory regions include:

- Hippocampus
- Entorhinal cortex
- Parahippocampal cortex
- Amygdala
- Fusiform gyrus
- Lingual gyrus
- Inferior/middle temporal cortex
- Anterior cingulum
- Posterior cingulum
- Precuneus
- Prefrontal cortex
- Superior temporal cortex
- Lateral parietal cortex
- Lateral occipital cortex
- Pericentral cortex
- Medial occipital cortex

11. PHARMACOKINETIC/PHARMACODYNAMIC DATA

11.1. Pharmacokinetic Sample Collection

Blood samples for plasma concentration of CT1812 will be collected at:

- Baseline V2 (Study Day 1)
- V3 (Study Day 15)
- V4 (Study Day 29)
- V5 (Study Day 43)
- V6 (Study Day 64)
- V7 (Study Day 85)
- V8 (Study Day 106)
- V9 (Study Day 127)
- V10 (Study Day 148)
- Final/Early Discontinuation V11 (Study Day 169) (for subjects participating in the Primary Study Only)
- V13 (Study Day 253) (for subjects participating in the Extension Study)
- Final/Early Discontinuation V15 (Study Day 337) (for subjects participating in the Extension Study)

CSF samples for concentration of CT1812 will be collected at:

- V1 (Study Day -60 to -1)/Step 4
- Final/Early Discontinuation V11 (Study Day 169) (for subjects participating in the Primary Study Only)
- V11 (Study Day 169) (for subjects participating in the Extension Study)
- Final/Early Discontinuation V15 (Study Day 337) (for subjects participating in the Extension Study)

11.2. Pharmacokinetic Concentrations

Plasma concentrations of CT1812 will be listed and plotted across collection days for each subject, and summarized with descriptive statistics by treatment group.

Plasma concentrations of CT1812 that are less than the limit of quantification (LOQ) of the assay will be designated a value of zero for the summation of concentration-time data.

Concentration data in listings will be presented to the same number of significant figures as provided by the analytical laboratory. Summary statistics will be displayed to the number of decimal places appropriate for the accuracy of the data.

Individual and summary pre-dose concentrations will be plotted across collection days with the plasma concentration axis displayed on a linear scale and on a logarithmic scale.

CSF concentrations will be listed for each subject, and summarized with descriptive statistics by dose level / treatment group. In addition, CSF and Plasma concentrations of CT1812 concentrations will be normalized on both a mg-dose and a mg/kg-dose basis to account for both dose and body weight. CT1812 CSF and plasma concentration will be time matched and the CT1812 CSF/plasma concentration ratios will be determined and summarized using descriptive statistics.

11.3. Pharmacokinetic Parameters

No pharmacokinetic parameters will be determined in this study.

11.4. Statistical Analysis of Pharmacokinetic Parameters

No pharmacokinetic parameters will be analyzed in this study.

11.5. Pharmacodynamic Endpoints

CSF samples will also be used for the evaluation of the following biomarkers associated with Alzheimer's disease:

- Aβ 40 monomer
- Aβ 42 monomer
- Tau
- phospho-tau
- neurogranin
- Synaptotagmin
- synpatosomal-associated protein-25 (SNAP25)
- NFL
- YKL-40 ELISA
- neuronal pentraxin-1
- neuronal pentraxin -2
- neuronal pentraxin receptor
- complexin-2
- syntaxin-1b
- sytaxin-7
- gamma-synuclein
- rab GDI alpha
- Phosphatidylethanolamine-binding protein 1
- AP-2 complex subunit beta
- 14-3-3 protein zeta/delta

CSF concentrations will be listed and plotted across collection days for each subject.

CSF concentrations less than the LOQ of the assay for pharmacodynamic analysis will be designated either a value of zero or of LOQ, as applicable, after clarification from the Sponsor.

11.6. Pharmacodynamic Analysis

Pharmacodynamic endpoints of CSF will be summarized by treatment and timepoint, using descriptive statistics for the original measurements as well as the change from baseline for continuous endpoints.

11.7. Statistical Analysis of Pharmacodynamic Parameters

Change from baseline in pharmacodynamic endpoints of CSF will be analysed using the MMRM or ANCOVA model described in section 9.3. The analysis will be performed for time points up to and including the 6-month time point, and 12-month time point respectively.

11.8. Changes from the Analysis Planned in the Protocol

There are no changes from the planned analysis described in the protocol.

12. DATA LISTINGS AND SUMMARY TABLES

12.1. Interim/Preliminary Data Analysis

No interim analyses are planned.

12.2. Listings and Tables for Clinical Study Report

The data listings and summary tables, and associated figures, planned to be generated from the study data are listed in Appendix 1 and Appendix 2, respectively. The numbering and titles may vary in the final presentation, depending on the amount of data to be presented.

Listings, tables and figures will be provided to the Sponsor in a form suitable for inclusion as appendices to the Clinical Study Report, in RTF or PDF format. The layout will be landscape A4 size, with a margin of 1 inch. The default font for listings and tables will be Courier New 8 pt.

In the summary tables other than those included in the main text of the report, special characters and formatting will not be used.

Data in listings will be ordered by cohort, then subject, then date/time. Screen failed subjects will be identified in the listings.

Data to be presented graphically includes: Concentration-time data for each subject, and mean concentration values at each nominal sampling time-point for plasma CT1812 concentration.

Mean values for total scores for cognitive and clinical outcomes (ADAS-Cog14, NTB, ADCS-ADL, MMSE, CDR-SB) will be presented graphically by treatment group over time.

12.3. Data Transfer to the Study Sponsor

At the conclusion of the study, Avance will provide the Sponsor with an electronic copy of the listings, tables and graphs. This may be provided by email or by upload, as determined by the Sponsor.

Details of the statistical analyses will be retained in the Avance study file for reference.

13. GENERAL CONSIDERATIONS FOR DATA MANAGEMENT AND ANALYSIS

13.1. Analysis Packages

SAS 9.4 or higher (SAS Institute Inc., Cary, NC, USA) will be used for generating data listings and summary tables and associated figures, and for performing statistical analysis.

13.2. Electronic Data Management

All listings, tables and figures for inclusion in the appendices of the clinical study report will be generated using SAS programs.

A copy of final listings, tables and figures will be retained in the Avance internal study file. A tracking log will be maintained detailing the date and initials of the staff member responsible for generating each listing, table or figure, and the staff member conducting the quality control review. Any erroneous results identified during subsequent checking process will be corrected by update of the SAS program, and the document recreated and then re-checked. To ensure an accurate data trail is maintained, corrections to electronic documents will be included on the tracking log, detailing the correction along with the date of the new version as well as the initials of the staff member responsible for the change and the verifying staff member. If statistical analysis is repeated with a modified data set, the new data produced will be saved as a new file.

13.3. Archiving

At the conclusion of the study, the final listings, tables and figures will be archived along with the analysis data sets and QC documentation. Copies will be retained in the Avance study file for a minimum of 15 years, and in the Avance secure archives in accordance with Avance standard operating procedures.

Appendix 1: Planned Data Listings

The following listings and figures of individual subject data are planned to be generated for this study, however the numbering and titles may vary, depending on the amount of data to be presented:

Listing/Figure #	Listings/Figure Title	Panel
**Subject Participation**		
Listing 16.2.1.1	Informed Consent	IC
Listing 16.2.1.2	Inclusion and Exclusion Criteria	IE
Listing 16.2.1.3	Screen Failure Information	SCF
Listing 16.2.1.4	Study Completion / Discontinuation	SCD
**Protocol Deviations**		
Listing 16.2.2.1	Protocol Deviations	DV
Listing 16.2.2.2	Additional Comments	CO
**Analysis Populations**		
Listing 16.2.3.1	Analysis Set Inclusion by Subject	(derived)
**Demographics and Baseline Information**		
Listing 16.2.4.1	Demographics	DM
Listing 16.2.4.2	Medical, Surgical and Psychiatric History	MH
Listing 16.2.4.3	Alzheimer’s Disease Diagnosis	AD
Listing 16.2.4.4	Physical Examination at Screening	PE
Listing 16.2.4.5	Serology Screening	SER
Listing 16.2.4.6	Height and Weight at Screening	HTWT
Listing 16.2.4.7	Blood sampling for Apolipoprotein E (ApoE) genotyping	APOE
Listing 16.2.4.8	Modified Hachinski exam	ACM
Listing 16.2.4.9	Geriatric Depression Score	ACM
**Treatment Administration**		
Listing 16.2.5.1	Study Drug Administration	EX
Listing 16.2.5.2	Pre-dose Eligibility Reviews	EL
**Pharmacokinetic, Pharmacodynamic and Efficacy Data**		
Listing 16.2.6.1	Pharmacokinetic Blood Sampling and Plasma Concentrations	PKB, Bioanalytical
Figure 16.2.6.2	Individual Plasma Concentrations Over Time (Linear)	Bioanalytical
Figure 16.2.6.3	Individual Plasma Concentrations Over Time (Semi-log)	Bioanalytical
Listing 16.2.6.4	Pharmacokinetic CSF Sampling and Concentrations	CSF, Bioanalytical
Listing 16.2.6.5	CT1812 CSF/plasma concentration ratio	Bioanalytical
Listing 16.2.6.6	Pharmacodynamic CSF Results	Bioanalytical
Figure 16.2.6.7	Individual Pharmacodynamic CSF Results Over Time	Bioanalytical
Listing 16.2.6.8	Brain Scan performed	BI

Listing/Figure #	Listings/Figure Title	Panel
Listing 16.2.6.8	Alzheimer's Disease Assessment Scale-Cognition Subscale (ADAS-Cog)	ADAS
Listing 16.2.6.9	Neuropsychological Test Battery (NTB)	NTB
Listing 16.2.6.10	Alzheimer's Disease Clinical Study – Activities of Daily Living (ADCS-ADL)	ADL
Listing 16.2.6.11	Mini Mental State Exam (MMSE)	MMSE, ACM
Listing 16.2.6.12	Clinical Dementia Rating Scale – Sum of the Boxes (CDR-SB)	CDR
Listing 16.2.6.13	Alzheimer's Disease Clinical Study – Clinician Global Impression of Change (ADCS-CGIC)	CGIC
Listing 16.2.6.14	Distribution Volume Ratio (DVR)	Imaging
Listing 16.2.6.15	SUV ratio (SUVR)	Imaging
Listing 16.2.6.16	Volumetric MRI	Imaging
Listing 16.2.6.17	Intrinsic Connectivity Contrast (ICC)	Imaging
**Safety Data (Adverse Events)**		
Listing 16.2.7.1	Adverse Events - CRF Data Only	AE, SAE
Listing 16.2.7.2	Adverse Events - MedDRA Coding and Calculated Values	AE, SAE
Listing 16.2.7.3	Prior and Concomitant Medications – CRF Data Only	CM
Listing 16.2.7.4	Prior and Concomitant Medications – WHODrug Coding and Calculated Values	CM
**Safety Data (Clinical Laboratory Evaluations)**		
Listing 16.2.8.1	Clinical Laboratory Blood Sample Collection	LB
Listing 16.2.8.2	Hematology Results	labdata
Listing 16.2.8.3	Clinical Chemistry Results	labdata
Listing 16.2.8.4	Coagulation Results	labdata
Listing 16.2.8.5	Thyroid Test Results	labdata
Listing 16.2.8.6	Clinical Laboratory Urine Sample Collection	LB
Listing 16.2.8.7	Urinalysis Results	labdata
**Safety Data (Other Assessments)**		
Listing 16.2.9.1	Vital Signs	VS
Listing 16.2.9.2	Vital Signs, Calculated Changes from Baseline	VS
Listing 16.2.9.3	ECG Parameters	EG
Listing 16.2.9.4	ECG Parameters, Calculated Changes from Baseline	EG
Listing 16.2.9.5	Height and Weight	HTWT
Listing 16.2.9.6	Physical Examinations Changes Over Time	PE
Listing 16.2.9.7	Neurological Examination	NE
Listing 16.2.9.8	Columbia Suicide Severity Rating Scale	CSSRS

Appendix 2: Planned Summary Tables

The following summary tables are planned to be produced, however the table numbers and titles may vary, depending on the amount of data to be presented:

14.1 Subject Data Summaries

- Table 14.1.1 Summary of Study Participation
- Table 14.1.2 Summary of Demographics
- Table 14.1.3 Summary of Treatment / Total Dose Administered

14.2 Pharmacokinetic and/or Efficacy Data Summaries

14.2.1 Pharmacokinetic Plasma Concentration Summaries

- Table 14.2.1.1 Summary of Plasma Concentrations of CT1812 Over Time by Treatment
- Figure 14.2.1.2 Mean Plasma Concentrations of CT1812 Over Time by Treatment (Linear)
- Figure 14.2.1.3 Mean Plasma Concentrations of CT1812 Over Time by Treatment (Semi-log)
- Table 14.2.1.4 Summary of Normalized Plasma Concentrations of CT1812 Over Time by Treatment

14.2.2 Pharmacokinetic CSF Concentration Summaries

- Table 14.2.2.1 Summary of CSF Concentrations Over Time by Treatment
- Table 14.2.2.2 Summary of Normalized CSF Concentrations Over Time by Treatment
- Table 14.2.2.3 CT1812 CSF/plasma concentration ratio

14.2.3 Pharmacodynamic Data Summaries

- Table 14.2.3.1 Summary of CSF biomarkers associated with Alzheimer's Disease by Treatment
- Figure 14.2.3.2 Mean Change from Baseline in CSF biomarkers associated with Alzheimer's Disease Over Time by Treatment
- Table 14.2.3.3 Analysis of Change from Baseline in CSF biomarkers associated with Alzheimer's Disease, up to 6-months
- Figure 14.2.3.4 LS Mean Change from Baseline in CSF biomarkers associated with Alzheimer's Disease Over Time by Treatment, up to 6-months

- Table 14.2.3.5 Analysis of Change from Baseline in CSF biomarkers associated with Alzheimer's Disease, up to 12-months
- Figure 14.2.3.6 LS Mean Change from Baseline in CSF biomarkers associated with Alzheimer's Disease Over Time by Treatment, up to 12-months

14.2.4 Efficacy Data Summaries

- Table 14.2.4.1 Individual Total Score for Alzheimer's Disease Assessment Scale-Cognition Subscales (ADAS-Cog11, ADAS-Cog13, ADAS-Cog14)
- Table 14.2.4.2 Summary of Total Score for Alzheimer's Disease Assessment Scale-Cognition Subscale (ADAS-Cog11)
- Figure 14.2.4.3 Mean Change from Baseline in Total Score for Alzheimer's Disease Assessment Scale-Cognition Subscale (ADAS-Cog11) Over Time
- Table 14.2.4.4 Analysis of Change from Baseline in Total Score for Alzheimer's Disease Assessment Scale-Cognition Subscale (ADAS-Cog11), up to 6-months
- Figure 14.2.4.5 LS Mean Change from Baseline in Total Score for Alzheimer's Disease Assessment Scale-Cognition Subscale (ADAS-Cog11) Over Time, up to 6-months
- Table 14.2.4.6 Analysis of Change from Baseline in Total Score for Alzheimer's Disease Assessment Scale-Cognition Subscale (ADAS-Cog11), up to 12-months
- Figure 14.2.4.7 LS Mean Change from Baseline in Total Score for Alzheimer's Disease Assessment Scale-Cognition Subscale (ADAS-Cog11) Over Time, up to 12-months
- Table 14.2.4.8 Summary of Total Score for Alzheimer's Disease Assessment Scale-Cognition Subscale (ADAS-Cog13)
- Figure 14.2.4.9 Mean Change from Baseline in Total Score for Alzheimer's Disease Assessment Scale-Cognition Subscale (ADAS-Cog13) Over Time
- Table 14.2.4.10 Analysis of Change from Baseline in Total Score for Alzheimer's Disease Assessment Scale-Cognition Subscale (ADAS-Cog13), up to 6-months
- Figure 14.2.4.11 LS Mean Change from Baseline in Total Score for Alzheimer's Disease Assessment Scale-Cognition Subscale (ADAS-Cog13) Over Time, up to 6-months
- Table 14.2.4.12 Analysis of Change from Baseline in Total Score for Alzheimer's Disease Assessment Scale-Cognition Subscale (ADAS-Cog13), up to 12-months

- Figure 14.2.4.13 LS Mean Change from Baseline in Total Score for Alzheimer's Disease Assessment Scale-Cognition Subscale (ADAS-Cog13) Over Time, up to 12-months
- Table 14.2.4.14 Summary of Total Score for Alzheimer's Disease Assessment Scale-Cognition Subscale (ADAS-Cog14)
- Figure 14.2.4.15 Mean Change from Baseline in Total Score for Alzheimer's Disease Assessment Scale-Cognition Subscale (ADAS-Cog14) Over Time
- Table 14.2.4.16 Analysis of Change from Baseline in Total Score for Alzheimer's Disease Assessment Scale-Cognition Subscale (ADAS-Cog14), up to 6-months
- Figure 14.2.4.17 LS Mean Change from Baseline in Total Score for Alzheimer's Disease Assessment Scale-Cognition Subscale (ADAS-Cog14) Over Time, up to 6-months
- Table 14.2.4.18 Analysis of Change from Baseline in Total Score for Alzheimer's Disease Assessment Scale-Cognition Subscale (ADAS-Cog14), up to 12-months
- Figure 14.2.4.19 LS Mean Change from Baseline in Total Score for Alzheimer's Disease Assessment Scale-Cognition Subscale (ADAS-Cog14) Over Time, up to 12-months
- Table 14.2.4.20 Individual Trail Making times for Neuropsychological Test Battery (NTB))
- Table 14.2.4.21 Summary of total completion time of Trail A and Trail B for Neuropsychological Test Battery (NTB)
- Figure 14.2.4.22 Mean Change from Baseline in total completion time of Trail A and Trail B for Neuropsychological Test Battery (NTB) Over Time
- Table 14.2.4.23 Analysis of total completion time of Trail A and Trail B for Neuropsychological Test Battery (NTB), up to 6-months
- Figure 14.2.4.24 LS Mean Change from Baseline in total completion time of Trail A and Trail B for Neuropsychological Test Battery (NTB) Over Time, up to 6-months
- Table 14.2.4.25 Analysis of total completion time of Trail A and Trail B for Neuropsychological Test Battery (NTB), up to 12-months
- Figure 14.2.4.26 LS Mean Change from Baseline in total completion time of Trail A and Trail B for Neuropsychological Test Battery (NTB) Over Time, up to 12-months
- Table 14.2.4.27 Individual Total Scores for Alzheimer’s Disease Clinical Study – Activities of Daily Living (ADCS-ADL)

- Table 14.2.4.28 Summary of Total Score for Alzheimer's Disease Clinical Study – Activities of Daily Living (ADCS-ADL)
- Figure 14.2.4.29 Mean Change from Baseline in Total Score for Alzheimer's Disease Clinical Study – Activities of Daily Living (ADCS-ADL) Over Time
- Table 14.2.4.30 Analysis of Change from Baseline in Total Score for Alzheimer's Disease Clinical Study – Activities of Daily Living (ADCS-ADL), up to 6-months
- Figure 14.2.4.31 LS Mean Change from Baseline in Total Score for Alzheimer's Disease Clinical Study – Activities of Daily Living (ADCS-ADL) Over Time, up to 6-months
- Table 14.2.4.32 Analysis of Change from Baseline in Total Score for Alzheimer's Disease Clinical Study – Activities of Daily Living (ADCS-ADL), up to 12-months
- Figure 14.2.4.33 LS Mean Change from Baseline in Total Score for Alzheimer's Disease Clinical Study – Activities of Daily Living (ADCS-ADL) Over Time, up to 12-months
- Table 14.2.4.34 Individual Total Scores for Mini Mental State Exam (MMSE)
- Table 14.2.4.35 Summary of Total Score for Mini Mental State Exam (MMSE)
- Figure 14.2.4.36 Mean Change from Baseline in Total Score for Mini Mental State Exam (MMSE) Over Time
- Table 14.2.4.37 Analysis of Change from Baseline in Total Score for Mini Mental State Exam (MMSE), up to 6-months
- Figure 14.2.4.38 LS Mean Change from Baseline in Total Score for Mini Mental State Exam (MMSE) Over Time, up to 6-months
- Table 14.2.4.39 Analysis of Change from Baseline in Total Score for Mini Mental State Exam (MMSE), up to 12-months
- Figure 14.2.4.40 LS Mean Change from Baseline in Total Score for Mini Mental State Exam (MMSE) Over Time, up to 12-months
- Table 14.2.4.41 Individual Total Scores for Clinical Dementia Rating Scale – Sum of the Boxes (CDR-SB)
- Table 14.2.4.42 Summary of Total Score for Clinical Dementia Rating Scale – Sum of the Boxes (CDR-SB)
- Figure 14.2.4.43 Mean Change from Baseline in Total Score for Clinical Dementia Rating Scale – Sum of the Boxes (CDR-SB) Over Time
- Table 14.2.4.44 Analysis of Change from Baseline in Total Score for Clinical Dementia Rating Scale – Sum of the Boxes (CDR-SB), up to 6-months

- Figure 14.2.4.45 LS Mean Change from Baseline in Total Score for Clinical Dementia Rating Scale – Sum of the Boxes (CDR-SB) Over Time, up to 6-months
- Table 14.2.4.46 Analysis of Change from Baseline in Total Score for Clinical Dementia Rating Scale – Sum of the Boxes (CDR-SB), up to 12-months
- Figure 14.2.4.47 LS Mean Change from Baseline in Total Score for Clinical Dementia Rating Scale – Sum of the Boxes (CDR-SB) Over Time, up to 12-months
- Table 14.2.4.48 Summary of Alzheimer's Disease Clinical Study – Clinician Global Impression of Change (ADCS-CGIC)
- Table 14.2.4.49 Analysis of Alzheimer's Disease Clinical Study – Clinician Global Impression of Change (ADCS-CGIC), up to 6-months
- Table 14.2.4.50 Analysis of Alzheimer's Disease Clinical Study – Clinician Global Impression of Change (ADCS-CGIC), up to 12-months
- Table 14.2.4.51 Frequency table for Alzheimer's Disease Clinical Study – Clinician Global Impression of Change (ADCS-CGIC)
- Table 14.2.4.52 Individual Scores for Composite z-score scales (Cognitive, NTB, Memory, Attention, Executive Function)
- Table 14.2.4.53 Summary of Scores for Composite z-score scales (Cognitive, NTB, Memory, Attention, Executive Function)
- Figure 14.2.4.54 Mean Change from Baseline in Scores for Composite z-score scales (Cognitive, NTB, Memory, Attention, Executive Function) Over Time
- Table 14.2.4.55 Analysis of Change from Baseline in Scores for Composite z-score scales (Cognitive, NTB, Memory, Attention, Executive Function), up to 6-months
- Figure 14.2.4.56 LS Mean Change from Baseline in Scores for Composite z-score scales (Cognitive, NTB, Memory, Attention, Executive Function) Over Time, up to 6-months
- Table 14.2.4.57 Analysis of Change from Baseline in Scores for Composite z-score scales (Cognitive, NTB, Memory, Attention, Executive Function), up to 12-months
- Figure 14.2.4.58 LS Mean Change from Baseline in Scores for Composite z-score scales (Cognitive, NTB, Memory, Attention, Executive Function) Over Time, up to 12-months
- Table 14.2.4.59 Correlation between CSF Biomarkers and Efficacy Scales at Baseline

- Table 14.2.4.60 Correlation between change from baseline in CSF Biomarkers and change from baseline in Efficacy Scales

14.2.5 Imaging Outcome Summaries

- Table 14.2.5.1 Summary of Distribution Volume Ratio (DVR)
- Table 14.2.5.2 Analysis of Change from Baseline in DVR: Composite region (pooled analysis and by arm), up to 6-months
- Table 14.2.5.3 Analysis of Change from Baseline in DVR: Exploratory regions (pooled analysis and by arm), up to 6-months
- Table 14.2.5.4 Analysis of Change from Baseline in DVR: Composite region (pooled analysis and by arm), up to 12-months
- Table 14.2.5.5 Analysis of Change from Baseline in DVR: Exploratory regions (pooled analysis and by arm), up to 12-months
- Table 14.2.5.6 Summary of SUV Ratio (SUVR)
- Table 14.2.5.7 Analysis of Change from Baseline in SUVR: Composite region (pooled analysis and by arm), up to 6-months
- Table 14.2.5.8 Analysis of Change from Baseline in SUVR: Exploratory regions (pooled analysis and by arm), up to 6-months
- Table 14.2.5.9 Analysis of Change from Baseline in SUVR: Composite region (pooled analysis and by arm), up to 12-months
- Table 14.2.5.10 Analysis of Change from Baseline in SUVR: Exploratory regions (pooled analysis and by arm), up to 12-months
- Table 14.2.5.11 Summary of Volumetric MRI
- Table 14.2.5.12 Analysis of Change from Baseline in Volumetric MRI: Composite region (pooled analysis and by arm), up to 6-months
- Table 14.2.5.13 Analysis of Change from Baseline in Volumetric MRI: Exploratory regions (pooled analysis and by arm), up to 6-months
- Table 14.2.5.14 Analysis of Change from Baseline in Volumetric MRI: Composite region (pooled analysis and by arm), up to 12-months
- Table 14.2.5.15 Analysis of Change from Baseline in Volumetric MRI: Exploratory regions (pooled analysis and by arm), up to 12-months
- Table 14.2.5.16 Summary of Intrinsic Connectivity Contrast (ICC)
- Table 14.2.5.17 Analysis of Change from Baseline in ICC: Composite region (pooled analysis and by arm), up to 6-months
- Table 14.2.5.18 Analysis of Change from Baseline in ICC: Exploratory regions (pooled analysis and by arm), up to 6-months

- Table 14.2.5.19 Analysis of Change from Baseline in ICC: Composite region (pooled analysis and by arm), up to 12-months
- Table 14.2.5.20 Analysis of Change from Baseline in ICC: Exploratory regions (pooled analysis and by arm), up to 12-months
- Table 14.2.5.21 Correlation between CSF Biomarkers and Imaging endpoints at Baseline
- Table 14.2.5.22 Correlation between change from baseline in CSF Biomarkers and change from baseline in Imaging endpoints
- Table 14.2.5.23 Correlation between Efficacy Scales and Imaging endpoints at Baseline
- Table 14.2.5.24 Correlation between change from baseline in Efficacy Scales and change from baseline in Imaging endpoints

14.3 Safety Data Summaries

14.3.1 Adverse Event Summaries

- Table 14.3.1.1 Overall Summary of Treatment-Emergent Adverse Events by Treatment
- Table 14.3.1.2 Summary of Treatment-Emergent Adverse Events by MedDRA Term and Treatment
- Table 14.3.1.3 Summary of Treatment-Emergent Adverse Events by Intensity (Severity) and Treatment
- Table 14.3.1.4 Summary of Treatment-Emergent Adverse Events by Relationship and Treatment
- Table 14.3.1.5 Summary of Treatment-Emergent Adverse Events of at least Moderate Intensity and Related to Study Treatment, by Treatment

14.3.2 Deaths, Other Serious and Significant Adverse Events

- Table 14.3.2.1 Subjects with Serious Adverse Events
- Table 14.3.2.2 Subjects with Adverse Events Leading to Study Withdrawal
- Table 14.3.2.3 Subjects Who Died During the Study

14.3.3 Narratives of Deaths, Other Serious and Certain Other Significant Adverse Event

<Narratives by Subject, to be provided by Safety Officer>

14.3.4 Clinical Laboratory Assessments

- Table 14.3.4.1 Summary of Hematology Results by Treatment
- Table 14.3.4.2 Summary of Clinical Chemistry Results by Treatment
- Table 14.3.4.3 Summary of Coagulation Results by Treatment

- Table 14.3.4.4 Summary of Assessments of Hematology Results by Treatment
- Table 14.3.4.5 Summary of Assessments of Clinical Chemistry Results by Treatment
- Table 14.3.4.6 Summary of Assessments of Coagulation Results by Treatment
- Table 14.3.4.7 Summary of Assessments of Urinalysis Results by Treatment
- Table 14.3.4.8 Individual Subject Profiles for Clinical Laboratory Parameters With at Least One Clinically Significant Result

14.3.5 Vital Signs

- Table 14.3.5.1 Summary of Vital Signs (Pulse Rate) by Treatment
- Table 14.3.5.2 Summary of Vital Signs (Systolic Blood Pressure) by Treatment
- Table 14.3.5.3 Summary of Vital Signs (Diastolic Blood Pressure) by Treatment
- Table 14.3.5.4 Summary of Vital Signs (Respiratory Rate) by Treatment
- Table 14.3.5.5 Summary of Vital Signs (Temperature) by Treatment
- Table 14.3.5.6 Summary of Vital Signs (Body weight) by Treatment

14.3.6 ECG Parameters

- Table 14.3.6.1 Summary of ECG Parameters (Ventricular Rate) by Treatment
- Table 14.3.6.2 Summary of ECG Parameters (PR Interval) by Treatment
- Table 14.3.6.3 Summary of ECG Parameters (QRS Duration) by Treatment
- Table 14.3.6.4 Summary of ECG Parameters (QT Interval) by Treatment
- Table 14.3.6.5 Summary of ECG Parameters (QTcF) by Treatment
- Table 14.2.6.6 Summary of ECG Parameters (QTcB) by Treatment
- Table 14.3.6.7 Summary of Derived Categorical QTcB by Treatment
