# Supplementary material for: A pilot study to evaluate the effect of CT1812 treatment on synaptic density and other biomarkers in Alzheimer’s disease
Source: Alzheimers Res Ther. 2024 Jan 25;16:20. doi: 10.1186/s13195-024-01382-2 (PMC10809445; doi:10.1186/s13195-024-01382-2)
Supplement: Supplementary file 2 — Additional file 2: Supplemental Table 1. Composite ROI of AD-affected brain regions. Supplemental Table 2. Exploratory brain regions. Supplemental Table 3. Volumetric MRI. Supplemental Table 4. CSF pharmacodynamic biomarkers. Supplemental Table 5. Safety including optional 6 month extension period (total 12 months). [file 13195_2024_1382_MOESM2_ESM.docx]

**SUPPLEMENTAL MATERIALS**

**Supplemental Table 1. Composite ROI of AD-affected brain regions**

| Cortical Region | Individual Region | Right Label Number | Left Label Number |
| --- | --- | --- | --- |
| **Medial Temporal** | Hippocampus | 53 | 17 |
|  | Entorhinal | 2006 | 1006 |
|  | Parahippocampal cortex | 2016 | 1016 |
|  | Amygdala | 54 | 18 |
| **Fusiform** | Fusiform gyrus | 2007 | 1007 |
| **Inf/Mid Temporal** | Inferior temporal gyrus | 2009 | 1009 |
|  | Middle temporal gyrus | 2015 | 1015 |
|  | Temporal pole | 2033 | 1033 |
| **Anterior Cingulum** | Rostral anterior cingulate cortex | 2026 | 1026 |
|  | Caudal anterior cingulate cortex | 2002 | 1002 |
| **Posterior Cingulum** | Posterior cingulate cortex | 2023 | 1023 |
|  | Isthmus of the cingulum | 2010 | 1010 |
| **Precuneus** | Precuneus | 2025 | 1025 |
| **Prefrontal** | Frontal pole | 2032 | 1032 |
|  | Superior frontal gyrus | 2028 | 1028 |
|  | Rostral middle frontal gyrus | 2027 | 1027 |
|  | Caudal middle frontal gyrus | 2003 | 1003 |
|  | Pars orbitals | 2019 | 1019 |
|  | Pars opercularis | 2018 | 1018 |
|  | Pars triangularis | 2020 | 1020 |
|  | Lateral orbitofrontal | 2012 | 1012 |
|  | Medal orbitofrontal | 2014 | 1014 |
| **Superior Temporal** | Banks of the superior temporal sulcus | 2001 | 1001 |
|  | Superior temporal gyrus | 2030 | 1030 |
|  | Transverse temporal gyrus | 2034 | 1034 |
| **Lateral Parietal** | Superior parietal lobule | 2029 | 1029 |
|  | Inferior parietal lobule | 2008 | 1008 |
|  | Supramarginal gyrus | 2031 | 1031 |
| **Lateral occipital** | Lateral occipital cortex | 2011 | 1011 |

The composite ROI of AD-affected regions included bilateral medial temporal, fusiform, inferior/middle temporal, anterior cingulum, posterior cingulum, precuneus, prefrontal, superior temporal, lateral parietal, and lateral occipital brain regions. Individual label (region) names are from the FreeSurfer [Version 6.0] Desikan-Killiany atlas and look up table for label numbers. For PET imaging outcomes, composite ROIs were calculated by averaging values of SV2A *DVR* and FDG *SUVR* from the above brain regions, weighted by volume. For MRI outcomes, composite ROI values were calculated as a sum of the volumes from the above brain regions, normalized to each participant’s estimated intracranial volume.

**Supplemental Table 2. Exploratory brain regions**

| Exploratory Region | Individual Region | Right Label Number | Left Label Number |
| --- | --- | --- | --- |
| **Hippocampus** | Hippocampus | 53 | 17 |
| **Entorhinal** | Entorhinal | 2006 | 1006 |
| **Parahippocampal** | Parahippocampal cortex | 2016 | 1016 |
| **Amygdala** | Amygdala | 54 | 18 |
| **Fusiform** | Fusiform gyrus | 2007 | 1007 |
| **Lingual** | Lingual gyrus | 2013 | 1013 |
| **Inf/Mid Temporal** | Inferior temporal gyrus | 2009 | 1009 |
|  | Middle temporal gyrus | 2015 | 1015 |
|  | Temporal pole | 2033 | 1033 |
| **Anterior Cingulum** | Rostral anterior cingulate cortex | 2026 | 1026 |
|  | Caudal anterior cingulate cortex | 2002 | 1002 |
| **Posterior Cingulum** | Posterior cingulate cortex | 2023 | 1023 |
|  | Isthmus of the cingulum | 2010 | 1010 |
| **Precuneus** | Precuneus | 2025 | 1025 |
| **Prefrontal** | Frontal pole | 2032 | 1032 |
|  | Superior frontal gyrus | 2028 | 1028 |
|  | Rostral middle frontal gyrus | 2027 | 1027 |
|  | Caudal middle frontal gyrus | 2003 | 1003 |
|  | Pars orbitals | 2019 | 1019 |
|  | Pars opercularis | 2018 | 1018 |
|  | Pars triangularis | 2020 | 1020 |
|  | Lateral orbitofrontal | 2012 | 1012 |
|  | Medal orbitofrontal | 2014 | 1014 |
| **Superior Temporal** | Banks of the superior temporal sulcus | 2001 | 1001 |
|  | Superior temporal gyrus | 2030 | 1030 |
|  | Transverse temporal gyrus | 2034 | 1034 |
| **Lateral parietal** | Superior parietal lobule | 2029 | 1029 |
|  | Inferior parietal lobule | 2008 | 1008 |
|  | Supramarginal gyrus | 2031 | 1031 |
| **Lateral Occipital** | Lateral occipital cortex | 2011 | 1011 |
| **Pericentral** | Paracentral gyrus | 2017 | 1017 |
|  | Postcentral gyrus | 2022 | 1022 |
|  | Precentral gyrus | 2024 | 1024 |
| **Medial Occipital** | Cuneus | 2005 | 1005 |
|  | Pericalcarine cortex | 2021 | 1021 |

Individual label (region) names are from the FreeSurfer [Version 6.0] Desikan-Killiany atlas and look up table for label numbers. For PET imaging outcomes, exploratory ROIs were calculated by averaging values of SV2A *DVR* and FDG *SUVR* from the above individual brain regions, weighted by volume. For MRI outcomes, exploratory ROIs were calculated as a sum of the volumes from the above individual brain regions, normalized to each participant’s estimated intracranial volume.

**Supplemental Table 3: Volumetric MRI**

|  | Placebo  (N=6) | CT1812 100 mg  (N=6) | CT1812 300 mg  (N=6) | Pooled  (N=12) |
| --- | --- | --- | --- | --- |
| LS Mean (SE) 24 Week Change from Baseline, mL  [95% CI from placebo; p-value] | | | | |
| Composite | -13.85 (3.39)  - | -5.59 (3.51)  [-2.01, 18.53; 0.11] | -6.34 (3.49)  [-2.40, 17.43; 0.13] | -5.94 (2.34)  [-0.50, 16.21; 0.06] |
| Hippocampus | -0.40 (0.11)  - | -0.19 (0.11)  [-0.11, 0.52; 0.19] | 0.02 (0.12)  [0.06, 0.77; 0.02*] | -0.09 (0.08)  [0.01, 0.57; 0.04*] |
| Entorhinal cortex | -0.08 (0.15)  - | -0.23 (0.15)  [-0.57, 0.28; 0.49] | 0.03 (0.15)  [-0.31, 0.54; 0.58] | -0.09 (0.10)  [-0.38, 0.37; 0.98] |
| Parahippocampal cortex | 0.13 (0.12)  - | 0.11 (0.12)  [-0.36, 0.32; 0.90] | -0.02 (0.12)  [-0.49, 0.19; 0.38] | 0.04 (0.08)  [-0.38, 0.21; 0.56] |
| Amygdala | -0.06 (0.07)  - | -0.04 (0.06)  [-0.16, 0.21; 0.77] | -0.04 (0.06)  [-0.17, 0.22; 0.80] | -0.04 (0.04)  [-0.14, 0.19; 0.76] |
| Fusiform gyrus | -1.00 (0.21)  - | -0.52 (0.21)  [-0.15, 1.10; 0.13] | -0.81 (0.21)  [-0.42, 0.79; 0.53] | -0.66 (0.16)  [-0.24, 0.88; 0.25] |
| Lingual gyrus | -0.19 (0.17)  - | -0.67 (0.17)  [-0.95, 0.01; 0.05] | -0.39 (0.17)  [-0.67, 0.29; 0.42] | -0.54 (0.12)  [-0.77, 0.10; 0.12] |
| Inferior/middle temporal cortex | -1.90 (0.54)  - | -1.15 (0.56)  [-0.89, 2.39; 0.36] | -1.14 (0.57)  [-0.84, 2.36;0.34] | -1.14 (0.38)  [-0.58, 2.09; 0.25] |
| Anterior cingulum | 0.01 (0.17)  - | -0.04 (0.17)  [-0.54, 0.43; 0.83] | -0.30 (0.18)  [-0.83, 0.21; 0.23] | -0.16 (0.12)  [-0.59, 0.26; 0.42] |
| Posterior cingulum | -0.34 (0.31)  - | -0.45 (0.31)  [-1.00, 0.78; 0.80] | -0.27 (0.32)  [-0.87, 1.01; 0.87] | -0.35 (0.22)  [-0.77, 0.78; 0.99] |
| Precuneus | -0.79 (0.26)  - | -0.39 (0.27)  [-0.39, 1.19; 0.31] | -0.49 (0.26)  [-0.45, 1.05; 0.42] | -0.44 (0.18)  [-0.31, 0.99; 0.29] |
| Prefrontal cortex | -4.80 (1.37)  - | 0.43 (1.41)  [1.20, 9.28; 0.01*] | -1.23 (1.39)  [-0.40, 7.55; 0.08] | -0.41 (0.95)  [1.01, 7.76; 0.01*] |
| Superior temporal cortex | -1.29 (0.41)  - | -0.87 (0.44)  [-0.86, 1.69; 0.51] | -0.31 (0.42)  [-0.21, 2.17; 0.10] | -0.58 (0.28)  [-0.29, 1.74; 0.16] |
| Lateral parietal cortex | -3.16 (0.81)  - | -1.30 (0.85)  [-0.70, 4.40; 0.15] | -1.35 (0.81)  [-0.52, 4.13; 0.12] | -1.33 (0.55)  [-0.19, 3.84; 0.07] |
| Lateral occipital cortex | -0.54 (0.22)  - | -0.98 (0.23)  [-1.10, 0.22; 0.18] | -0.36 (0.22)  [-0.46, 0.82; 0.57] | -0.67 (0.16)  [-0.70, 0.46; 0.67] |
| Pericentral cortex | -2.90 (0.77)  - | 0.26 (0.78)  [0.89, 5.42; 0.01*] | -0.11 (0.77)  [0.56, 5.01; 0.02*] | 0.07 (0.53)  [1.08, 4.85; 0.00*] |
| Medial occipital cortex | -0.49 (0.22)  - | -0.42 (0.22)  [-0.57, 0.70; 0.84] | -0.08 (0.22)  [-0.23, 1.04; 0.21] | -0.25 (0.15)  [-0.31, 0.78; 0.39] |
| Lateral ventricle | 11.47 (6.13)  - | -3.68 (6.15)  [-33.08, 2.77; 0.09] | 4.49 (6.38)  [-25.34, 11.38; 0.44] | 0.37 (4.33)  [-26.43, 4.16; 0.15] |
| eICV | -3.79 (4.26)  - | -6.70 (4.40)  [-15.43, 9.61; 0.64] | -8.85 (4.30)  [-17.40, 7.29; 0.41] | -7.78 (2.92)  [-14.33, 6.32; 0.44] |
| Cerebral cortex | -17.45 (4.28)  - | -9.70 (4.41)  [-5.06, 20.57; 0.23] | -6.20 (4.34)  [-1.17, 23.68; 0.07] | -7.88 (2.95)  [-0.88, 20.12; 0.07] |

**Supplemental Table 4: CSF pharmacodynamic biomarkers**

|  | Placebo  (N=6) | CT1812 100 mg  (N=5) | CT1812 300 mg  (N=7) | Pooled  (N=12) |
| --- | --- | --- | --- | --- |
| LS Mean (SE) 24 Week Change from Baseline  [95% CI for LS Mean Change from Baseline; p-value] | | | | |
| Aβ40, pg/ml | 222.25 (397.27)  [-629.8, 1074.3; 0.58] | 517.70 (445.24)  [-437.2, 1472.7; 0.26] | -594.0 (381.57)  [-1412, 224.38); 0.14] | -118.1 (301.49)  [-760.7, 524.55; 0.70] |
| Aβ42, pg/ml | -13.90 (19.66)  [-56.07, 28.27; 0.49] | 11.62 (21.88)  [-35.30, 58.55; 0.60] | -32.96 (17.55)  [-70.60, 4.69; 0.08] | -15.46 (14.31)  [-45.95, 15.03; 0.30] |
| Tau, pg/ml | 36.74 (120.70)  [-222.1, 295.62; 0.77] | 121.22 (131.96)  [-161.8, 404.26; 0.37] | -84.08 (111.96)  [-324.2, 156.06; 0.47] | 1.90 (86.38)  [-182.2, 186.01; 0.98] |
| pTau, pg/ml | -4.67 (13.59)  [-33.81, 24.48; 0.74] | 5.80 (14.91)  [-26.17, 37.77; 0.70] | -7.71 (12.60)  [-34.75, 19.32; 0.55] | -2.08 (9.44)  [-22.19, 18.04; 0.83] |
| Neurogranin, fmol/µL | -0.01 (0.01) [-0.03, 0.00; 0.16] | 0.00 (0.01)  [-0.01, 0.02; 0.96] | -0.01 (0.01)  [(-0.02, 0.01; 0.22] | -0.00 (0.00)  [-0.01, 0.01; 0.35] |
| Synaptotagmin, pM | 0.68 (2.44)  [-4.55, 5.91; 0.78] | 6.37 (2.64)  [0.72, 12.03; 0.03] | 0.88 (2.24)  [-3.93, 5.69; 0.70] | 3.18 (1.80)  [-0.65, 7.01; 0.10] |
| SNAP-25, pM | 1.36 (1.66)  [-2.21, 4.92; 0.43] | 0.74 (1.83)  [(-3.19, 4.68; 0.69] | -3.15 (1.54)  [-6.45, 0.15; 0.06] | -1.54 (1.24)  [-4.17, 1.10; 0.23] |
| NFL, pg/ml | -24.02 (164.56)  [-377.0, 328.93; 0.89] | 265.74 (176.40)  [-112.6, 644.08; 0.15] | 210.77 (149.24)  [-109.3, 530.86; 0.18] | 233.64 (111.08)  [-3.12, 470.39; 0.05] |

**Supplemental Table 5: Safety including optional 6 month extension period (total 12 months)**

|  | Placebo  (N=7) | CT1812 100 mg  (N=8) | CT1812 300 mg  (N=8) | Total  (N=23) |
| --- | --- | --- | --- | --- |
| Number (%) of Subjects with TEAE  [Number of TEAEs] | | | | |
| TEAEs | 6 (86%)  [11] | 8 (100%)  [42] | 7 (88%)  [23] | 21 (91%)  [76] |
| Mild | 4 (57%)  [9] | 4 (50%)  [37] | 6 (75%)  [19] | 14 (61%)  [65] |
| Moderate | 1 (14%)  [1] | 2 (25%)  [3] | 1 (13%)  [4] | 4 (17%)  [8] |
| Severe | 1 (14%)  [1] | 2 (25%)  [2] | 0 (0%)  - | 3 (13%)  [3] |
| Related TEAEs | 4 (57%)  [4] | 3 (38%)  [3] | 4 (50%)  [7] | 11 (48%)  [14] |
| TEAEs leading to treatment discontinuation | 1 (14%)  [1] | 2 (25%)  [2] | 2 (25%)  [2] | 5 (22%)  [5] |
| SAEs | 1 (14%)  [1] | 3 (38%)  [4] | 0 (0%)  - | 4 (17%)  [5] |
| Related SAEs | 0 (0%) | 0 (0%) | 0 (0%) | 0 (0%) |
| Number (%) of Subjects with Treatment-Related* TEAE  [Number of TEAEs] | | | | |
| Headache | 2 (29%)  [2] | 2 (25%)  [2] | 3 (38%)  [3] | 7 (30%)  [7] |
| Dizziness | 0 (0%) | 1 (13%)  [1] | 1 (13%)  [3] | 2 (9%)  [4] |
| Liver function test increase | 0 (0%) | 0 (0%) | 2 (25%)  [2] | 2 (9%)  [2] |
| Vomiting | 1 (14%)  [1] | 2 (25%)  [2] | 0 (0%) | 3 (13%)  [3] |
| Diarrhea | 1 (14%)  [1] | 0 (0%) | 0 (0%) | 1 (4%)  [1] |
| Total related TEAEs | 4 (57%)  [4] | 3 (38%)  [3] | 4 (50%)  [7] | 10 (43%)  [13] |

Top: Number and percentage of subjects experiencing an event and the total number of events in brackets for the combined six-month primary study period and optional six-month extension are summarized. The denominator for percentage corresponds to the N in each column. N is the number of subjects in the Safety Analysis Set. Bottom: AEs were coded using MedDRA version 21.0. TEAEs are events that occurred or worsened on or after the first application of study drug. Subjects are counted only once for each system organ class (SOC) and once for each preferred term (PT). The severity shown is the greatest severity reported for a particular subject. TEAE: treatment-emergent adverse event; SAE: severe adverse event; *Related = possibly, probably, or definitely related.
